# *Polydomus karssenii* gen. nov. sp. nov. is a dark septate endophyte with a bifunctional lifestyle parasitising eggs of plant parasitic cyst nematodes (*Heterodera* spp.)

**DOI:** 10.1186/s43008-023-00113-w

**Published:** 2023-03-30

**Authors:** Samad Ashrafi, Jan-Peer Wennrich, Yvonne Becker, Jose G. Maciá-Vicente, Anke Brißke-Rode, Matthias Daub, Torsten Thünen, Abdelfattah A. Dababat, Maria R. Finckh, Marc Stadler, Wolfgang Maier

**Affiliations:** 1 Institute for Epidemiology and Pathogen Diagnostics, Julius Kühn Institute (JKI) – Federal Research Centre for Cultivated Plants, Messeweg 11/12, 38104 Brunswick, Germany; 2 Institute for Crop and Soil Science, Julius Kühn Institute (JKI) – Federal Research Centre for Cultivated Plants, Bundesallee 58, 38116 Brunswick, Germany; 3 Department Microbial Drugs, Helmholtz Centre for Infection Research, Inhoffenstraße 7, 38124 Brunswick, Germany; 4 Institute of Microbiology, Technische Universität Braunschweig, Spielmannstraße 7, 38106 Brunswick, Germany; 5 Plant Ecology and Nature Conservation, Wageningen University and Research, PO Box 47, 6700 AA Wageningen, The Netherlands; 6 Institute for Plant Protection in Field Crops and Grassland, Julius Kühn Institute (JKI) – Federal Research Centre for Cultivated Plants, Dürener Str. 71, 50189 Elsdorf, Germany; 7 International Maize and Wheat Improvement Centre (CIMMYT), Emek, P.O. Box 39, 06511 Ankara, Turkey; 8 Department of Ecological Plant Protection, University of Kassel, Witzenhausen, Germany

**Keywords:** Endophytes, Nematophagous fungi, New species, Phylogeny, Plant parasitic nematodes, Taxonomy

## Abstract

In this study fungal strains were investigated, which had been isolated from eggs of the cereal cyst nematode *Heterodera filipjevi*, and roots of *Microthlaspi perfoliatum* (*Brassicaceae*). The morphology, the interaction with nematodes and plants and the phylogenetic relationships of these strains originating from a broad geographic range covering Western Europe to Asia Minor were studied. Phylogenetic analyses using five genomic loci including ITSrDNA, LSUrDNA, SSUrDNA, *rpb2* and *tef1-α* were carried out. The strains were found to represent a distinct phylogenetic lineage most closely related to *Equiseticola* and *Ophiosphaerella*, and *Polydomus karssenii* (*Phaeosphaeriaceae*, *Pleosporales*) is introduced here as a new species representing a monotypic genus. The pathogenicity tests against nematode eggs fulfilled Koch’s postulates using in vitro nematode bioassays and showed that the fungus could parasitise its original nematode host *H*. *filipjevi* as well as the sugar beet cyst nematode *H*. *schachtii*, and colonise cysts and eggs of its hosts by forming highly melanised moniliform hyphae. Light microscopic observations on fungus-root interactions in an axenic system revealed the capacity of the same fungal strain to colonise the roots of wheat and produce melanised hyphae and microsclerotia-like structure typical for dark septate endophytes. Confocal laser scanning microscopy further demonstrated that the fungus colonised the root cells by predominant intercellular growth of hyphae, and frequent formation of appressorium-like as well as penetration peg-like structures through internal cell walls surrounded by callosic papilla-like structures. Different strains of the new fungus produced a nearly identical set of secondary metabolites with various biological activities including nematicidal effects irrespective of their origin from plants or nematodes.

**Supplementary Information:**

The online version contains supplementary material available at 10.1186/s43008-023-00113-w.

## INTRODUCTION

Among root endophytic fungi, dark septate endophytes (DSE) represent a non-clavicipitaceous group with likely benefits for plant growth and health (Rodriguez et al. 2009). They constitute a phylogenetically diverse, polyphyletic group of fungi that share root-colonising habits and morphological traits, including formation of melanised inter- or intracellular hyphae, and development of microsclerotia within plant roots (Jumpponen and Trappe 1998). They are globally distributed across terrestrial ecosystems and plant lineages (Mandyam and Jumpponen 2005). Their ecological functions are still poorly understood, and are ranging from mutualistic to pathogenic (Ruotsalainen et al. 2022) but with frequently unknown effects for plant fitness. As mutualistic symbionts, they have been shown to be involved in nitrogen translocation to the host plants (Usuki and Narisawa 2007), alleviation of abiotic stresses such as drought and salinity (Gonzalez Mateu et al. 2020; Li et al. 2019; Usuki and Narisawa 2007), and tolerance to biotic stresses such as plant pathogens and pests (Su et al. 2013). Endophytic fungi (Rodriguez et al. 2009) in general are promising candidates for biological-based control approaches due to their diversity, wide distribution and multifunctional lifestyles (Latz et al. 2018; Schouten 2016). They can suppress plant pathogens through different mechanisms (Latz et al. 2018). For example, the endophytic insect-pathogenic fungi, including species in the genera *Beauveria* and *Metarhizium,* antagonise plant pests and translocate insect-derived nitrogen from parasitised insect cadavers to the plant host via fungal mycelia (Behie et al. 2012) in exchange for carbon provided by the plant (Behie et al. 2017). This specific tripartite interaction has provided insights into the cellular and molecular processes controlling this antagonistic-mutualistic symbiosis (Barelli et al. 2016; Hu and Bidochka 2021).

Plant parasitic nematodes (PPNs) attack their hosts mostly via root systems (Jones et al. 2013). They are associated with most terrestrial plants via a wide range of interactions, by which they acquire nutrients. Cyst forming nematodes (CNs), in particular *Heterodera* and *Globodera* spp., are among the most destructive plant parasitic nematodes due to their complex biotrophic parasitism and multiple developmental stages (Jones et al. 2013). They are sedentary parasites and establish highly specialised feeding sites (syncytia) by manipulating the physiology of the roots of host plants. The sedentary nature of cyst nematodes in plant tissues however makes them vulnerable to invasion by natural enemies present in the rhizosphere (Lopez-Llorca et al. 2008). Cyst nematodes were the first group of PPNs documented to be colonised by nematode parasitic fungi (Kühn 1877). Since then, a great diversity of fungi has been reported as nematophagous fungi, a functional group of fungal species capable of parasitising and feeding on nematodes (Hsueh et al. 2013; Stirling 2014). Among these fungi, several fungal species colonise eggs of different PPNs including cyst and root knot nematodes (*Meloidogyne* spp.) and are known as egg-parasitic nematophagous fungi (Nordbring-Hertz et al. 2011; Tribe 1977).

We have recently reported *Polyphilus sieberi* as the first dark septate endophytic fungus also parasitising the eggs of a cereal cyst nematode (CCN) (Ashrafi et al. 2018). The fungus was isolated from symptomatic cysts of *Heterodera filipjevi* Madzhidov collected from Turkey. Other strains of the fungus had also been isolated from the roots of various species of grasses and bushes in the Hungarian steppe, and phylogenetic identity of all isolates including nematode and plant derived strains was confirmed using multi-gene phylogenetic analyses (Ashrafi et al. 2018). During the same survey, in which *P. sieberi* was isolated from nematode eggs, symptomatic cysts exhibiting unusual discolouration and fungal colonisation were additionally scrutinised for further nematode egg parasitic fungi. Two strains DSM106825 (YE1) and DSM111209 (5BD) were found to be representing an undescribed species of the order *Pleosporales*. These strains also showed a high ITS sequence similarity with other undescribed pleosporalean fungal isolates reported as endophytes in roots of the plant species *Microthlaspi perfoliatum* (L.) F.K. Meyer (*Brassicaceae*) (Glynou et al. 2016). The plant derived strains included P1597, P2789, P2870, and P6040. While screening these strains for antimicrobial secondary metabolites (Helaly et al. 2018a) we also initially evaluated the phylogenetic relationship of the studied strains and found that they formed a monophyletic lineage within the family of *Phaeosphaeriaceae* (*Pleosporales*, *Dothideomycetes*) (cf. Supplementary Information in Helaly et al. (2018a)). To confirm these preliminary results, here we further studied the isolates from *H. filipjevi* and *M. perfoliatum* to: (i) determine the taxonomic novelty of the nematode and plant-derived strains and to study their phylogenetic placement in greater detail; (ii) examine the in vitro antagonistic associations of the nematode isolated strains with nematode eggs; (iii) provide microscopic observations on the endophytic interaction of the fungus with wheat (*Triticum aestivum*) as the host of *H. filipjevi*; and (iv) to compare the secondary metabolite profiles of the plant versus nematode isolated strains.

## MATERIAL AND METHODS

### Material examined and fungal isolation

The fungal strains studied here were either isolated from the CCN *H. filipjevi* or the plant species *M. perfoliatum* (Table 1). The nematode-associated strains were isolated from fungus-infected eggs of *H. filipjevi* that were collected from experimental wheat fields of CIMMYT (International Maize and Wheat Improvement Centre) naturally infested with nematodes located in Yozgat (39.08 N, 34.10 E; altitude, 985 m.a.s.l) in the Central Anatolian Plateau of Turkey in 2013. The details of nematode sampling are provided in Helaly et al. (2018a). Nematode cysts were extracted from soil and processed for fungal isolation as previously described (Ashrafi et al. 2017a). Briefly, cysts were surface disinfected using 0.5% (v/v) sodium hypochlorite (NaOCl) for 10 min. The disinfected cysts were cut open and the eggs showing symptoms of fungal infection or discolouration were surface-disinfected in NaOCl 0.5% for up to 3 min, followed by six rinses with sterilised distilled water. The eggs were then plated on potato dextrose agar (PDA; Merck, Germany) or cornmeal agar (CMA; Sigma Aldrich, Missouri, USA) and incubated at room temperature for fungal growth.

**Table 1:** List of fungal strains examined in this study

Strain	Isolate number	Source of isolate	Country of origin/locality	References
YE1	DSM106825 = JKI 72994	Eggs of *Heterodera filipjevi*	Turkey 39.08 N, 34.10 E	Helaly et al. (2018a)
5BD	DSM111209 = JKI 73116	Eggs of *Heterodera filipjevi*	Turkey 39.08 N, 34.10 E	This study
P1597	DSM111342 = JKI 73117	Roots of *Microthlaspi perfoliatum*	Bulgaria 42.66 N, 22.81 E	Glynou et al. (2016)
P2789	JKI 73120	Roots of *Microthlaspi perfoliatum*	Germany 48.55N, 10.12 E	Glynou et al. (2016)
P2870	JKI 73119	Roots of *Microthlaspi perfoliatum*	Germany 49.27N, 9.84 E	Glynou et al. (2016)
P6108	JKI 73118	Roots of *Microthlaspi perfoliatum*	Germany 49.54N, 9.34 E	Glynou et al. (2016)

DSM: The open collection of the Leibniz-Institut DSMZ- German Collection of Microorganisms and Cell Cultures GmbH; JKI: Julius Kühn Institute culture collection, Mykothek

The fungal strains originating from plant roots were isolated in 2013 from *M. perfoliatum* plants collected at various geographical locations in Germany and Bulgaria (Glynou et al. 2016). Roots of the plants were disinfected using 0.5% sodium hypochlorite for 1 min and then rinsed three times using sterile deionised water. Further processing for fungal isolation was as detailed in Glynou et al. (2016).

### Growth rate assays

The growth rates of fungal strains were determined on different agar media at 5 °C intervals of temperatures ranging between 5 and 35 °C in the dark. Various culture plates were inoculated by placing 4-mm diam. agar disks excised from cultures freshly grown on PDA. The colony growth was measured weekly for a 4-week period.

Fungal sporulation was examined on various culture media under different incubation conditions. Strains were grown on PDA, one-third or one-sixth strength PDA (PDA 1/3, PDA 1/6), CMA, CaCO_3_ agar [30 g CaCO_3_, 15 g agar, deionised water 1L, pH 7.4; (Su et al. 2012)], Czapek Dox agar (CZA; Sigma Aldrich, Missouri, USA), malt extract agar (MEA; Roth; Germany), modified Melin-Norkrans medium (MMN; Plantmedia, Ohio, USA), oatmeal agar (OA; Sigma Aldrich, Missouri, USA), and synthetic nutrient-poor agar [SNA; (Nirenberg 1976)] in the dark and in a 12 h/12 h light–dark rhythm, at 5 to 35 °C at intervals of 5 °C. The cultures were also incubated at 20 °C in a 12 h/12 h cycle of black light/darkness for up to 12 months. Another set of the cultures was supplemented with autoclaved wheat straw and examined for sporulation under the previously mentioned conditions. The third set of cultures was incubated at ambient conditions for a period of 1 year. Additionally, agar plugs of fungal cultures were incubated in sterilised deionised water (SDW) at room temperature, and at 10 and 20 °C in the dark for 6 months.

### Metabolite profiling

#### Cultivation and extraction of submerged cultures

To screen fungal strains for secondary metabolites, submerged cultures were prepared in 500 mL Erlenmeyer flasks using 200 mL of three different media: Q6/2 (10 g glycerol, 5 g cotton seed flour, 2.5 g d-glucose, deionised water 1 L, pH 7.2) YM 6.3 (10 g malt extract, 4 d-glucose, 4 g yeast extract, deionised water 1 L, pH 6.3) and ZM/2 media (5 g molasses, 5 g oatmeal, 4 g sucrose, 4 g mannitol, 1.5 g d-glucose, 1.5 g CaCO_3_, 0.5 g edamin K (lactalbumin hydrolysate), 0.5 g (NH_4_)_2_SO_4_, deionised water 1 L, pH 7.2). Strains were subcultured on YM 6.3 agar medium. A seed culture of 40 mL of Q6/2 was inoculated with few 5-mm-diameter culture discs of the cultures obtained from YM 6.3 agar. The submerged cultures were incubated for several days at 140 rpm and 23 °C on a rotary shaker until sufficient amount of mycelia were grown for subsequent homogenization. Screening cultures were inoculated with 0.5% of the homogenized seed culture. Incubation was performed with the same conditions mentioned above until 2 days after depletion of free glucose. Mycelia and supernatant were segregated by filtration and extracted as previously described (Becker et al. 2020).

#### Analysis of crude extracts

For HPLC–DAD/MS analysis, the crude extracts were diluted to 4.5 mg/mL in acetone/methanol (1:1) and 2 µL were injected to an UltiMate® 3000 Series uHPLC (Thermo Fisher Scientific, Waltman, MA/USA) using a C18 Acquity® UPLC BEH column (2.1 × 50 mm, 1.7 µm; Waters, Milford, MA/USA). HPLC was performed with the following settings: solvent A: H_2_O + 0.1% formic acid, solvent B: acetonitrile + 0.1% formic acid; gradient: 5% B (0.5 min), 5–100% (19.5 min), 100% (5 min), flowrate 0.6 mL/min, and DAD detection 190–600 nm. Mass spectrometry was performed with a connected amaZon® speed ESI Iontrap MS (Bruker).

### DNA extraction, PCR amplification and sequencing

Genomic DNA was extracted from fungal mycelia of the pure cultures using a modified cetyl trimethylammonium bromide (CTAB) method (Ashrafi et al. 2017a; Saghai-Maroof et al. 1984). Five nuclear loci were amplified using polymerase chain reaction (PCR): the internal transcribed spacers including the 5.8S rDNA (ITS) using the primers ITS1F (Gardes and Bruns 1993) and ITS4 (White et al. 1990); the partial large subunit of the ribosomal RNA (LSU rRNA), using the primer pair LR0R (Rehner and Samuels 1994) and LR5 (Vilgalys and Hester 1990); the partial small subunit of the ribosomal RNA (SSU rRNA), using the primers NS1 and NS4 (White et al. 1990); the partial RNA polymerase II second-largest subunit (*rpb2*) with the primer pairs rpb2F and rpb2R (Flores et al. 2017) or fRPB2-5F and fRPB2-7cR (Liu et al. 1999); and the partial translation-elongation factor 1-α (*tef1*) using the primers EF1-983f and EF1-2218r (Rehner 2001). PCR for the ITS, LSU, and *tef1* was performed as described previously (Ashrafi et al. 2017b). The *rpb2* and SSU regions were amplified in a final volume of 25 µl containing 1 µm template DNA, 12.5 µl of ALLin Hot Start Taq Mastermix, 2X (highQu ALLin™, Hot Start Taq Mastermix), 2 μl of each primer (0.4 pM μl^−1^), and DNA-free H_2_O. PCR reactions were carried out in a T-GRADIENT thermocycler (Biometra, Göttingen, Germany) with the thermal cycle programmes as follows: initial denaturation at 95 °C (2 min) followed by 35 cycles of denaturation at 95 °C (15 s), annealing at 50 °C (SSU), or 60 °C (*rpb2*) (15 s), extension at 72 °C (15 s), and a final extension at 72 °C (10 min). PCR products were purified using the DNA Clean & Concentrator™-5 kit (Zymo Research Corp., Irvine, California, USA) and sequenced by Eurofins Genomics GmbH, (Ebersberg, Germany) with the same primers used for PCR amplification. The sequences obtained were assembled as contigs, control-read, edited and trimmed with Sequencher 5.4.1 (Gene Codes Corporation, Ann Arbor, Michigan, USA) and deposited in GenBank under the following accession numbers: ON407111–ON407121 (ITS), ON407074–ON407084 (LSU), ON408345–ON408355 (SSU), ON419499–ON419509 (*tef1*), ON419510–ON419520 (*rpb2*). The sequences obtained were compared to those of publicly available using BLASTn searches within GenBank (http://blast.ncbi.nlm.nih.gov/Blast.cgi) (Altschul et al. 1990).

### Sequence comparison and phylogenetic analyses

The newly generated DNA sequences were combined with those from a previous dataset (Helaly et al. 2018a) and other sequences publicly available at NCBI GenBank, originating from representative specimens of *Phaeosphaeriaceae* following Mapook et al. (2020), Phookamsak et al. (2014), Phookamsak et al. (2017), Tennakoon et al. (2020), and Yuan et al. (2020a). The sequences were aligned using the online version of Mafft v.7 (https://mafft.cbrc.jp/alignment/server/) (Katoh et al. 2019; Kuraku et al. 2013) adopting the iterative refinement methods FFT-INS-i for the sequences of ITS, and LSU, and L-INS-i for those of SSU, *rpb2* and *tef1*. Alignments were refined manually if needed and the starts and ends of the sequences were trimmed using AliView (Larsson 2014), if needed.

Two datasets were created for the phylogenetic analyses: A dataset including sequences of four loci (ITS, LSU, SSU, and *tefA*) of 112 sequences representing 101 taxa to place the studied strains within this pleosporalean family; and a five-loci dataset (ITS, LSU, SSU, *rpb2* and *tefA*) from a subset of the taxa in the previous dataset (Additional file 1: Table S1), to provide better phylogentic resolutions for the taxa closely related to the focal strains.

Multigene phylogenetic analyses were applied using Bayesian inference (BI), maximum-likelihood (ML) and neighbor-joining (NJ). Bayesian analysis was performed using Metropolis Coupled Monte Carlo Markov chains implemented in MrBayes v3.2 (Huelsenbeck and Ronquist 2001; Ronquist and Huelsenbeck 2003). Evolutionary models for the Bayesian phylogenetic analyses were selected independently for each dataset using MrModeltest v. 3 under the hierarchial likelihood ratio test (hLRT) and the Akaike Information Criterion (AIC). The general time reversible model with gamma distributed substitution rates and invariant sites (GTR + I + G) was selected as the best fitting model for each individual data set and was implemented for the BI analyses accordingly. The process was run for 5,000,000 and 1,000,000 generations and trees were sampled every 500th and 100th generations for the four-loci and five-loci datasets, respectively. A 50% majority rule consensus tree was computed only from trees of the plateau, and if, additionally, the split frequencies were below 0.01. Thus, the trees representing the “burn-in phase” were discarded and the remaining trees were used to infer posterior probabilities (PP) for the nodes of the majority rule consensus tree.

For the larger dataset (four-loci dataset), maximum likelihood analyses were performed on the web server of IQ-Tree2 (http://www.iqtree.org/) (Minh et al. 2020; Nguyen et al. 2015) following the default parameters, the best-fitting substitution model and Ultrafast Bootstrap (1000) (Hoang et al. 2017). For the five-loci dataset, maximum likelihood (ML) phylogenetic analyses were carried out using RAxML 7.2.8 (Silvestro and Michalak 2012; Stamatakis 2014) as implemented in Geneious 8.1.2 applying the general time-reversible (GTR) substitution model with gamma model of rate heterogeneity (GTR + G) and 1000 replicates of rapid bootstrapping and search for best-scoring ML tree, starting with a complete random tree. The neighbor-joining analysis (Saitou and Nei 1987) was done in PAUP 4.0a in the batch file mode (Swofford 2002) applying the Kimura two-parameter model of DNA substitution (Kimura 1980) with a transition/transversion ratio of 2.0 to compute genetic distances. The phylogenetic trees were visualized using FigTree v1.4.3 (http://tree.bio.ed.ac.uk/software/figtree) and annotated using Adobe Illustrator CS 5.1. The final alignment and trees were deposited at Figshare repository (10.6084/m9.figshare.21558846).

### Light and confocal laser-scanning microscopy

#### Morphological examination

Fungal structures and fungus-infected nematode eggs were examined and photographed using a Zeiss Axioskop 2 plus compound microscope (Göttingen, Germany) and an Olympus SZX 12 stereo microscope (Tokyo, Japan) equipped with a Jenoptik ProgRes® digital camera. Images were recorded using CapturePro 2.8 software (Jenoptic, Jena, Germany). Growing mycelia and fungus-infected eggs were mounted in water or in slide cultures (Gams et al. 1998) and photographed. Nematode cysts were photographed in water in a square cavity dish (40 × 40 × 16 mm). To illustrate fungal colonisation of nematode eggs in vitro, slide cultures were prepared and then photographed as described in Ashrafi et al. (2017a). Microscopic specimens were studied using Differential Interference Contrast optics. The brightness and contrast of micrographs were further adjusted using Adobe Photoshop software CS 5.1. Colour codes used in the description were determined according to https://www.ral-farben.de/en/all-ral-colours.

#### Pathogenicity tests against cyst nematodes

To fulfil Koch’s postulates, in vitro tests were performed, where the parasitic nature of the strain DSM 106825 was examined against the CCN *H. filipjevi* as the origin of this isolate. To further assess the pathogenicity of the fungus against other cyst nematodes, strain DSM 106825 was also tested against the sugar beet cyst nematode (SBN) *Heterodera schachtii*. For these studies the cysts and eggs of the CCN *H. filipjevi* and the sugar beet cyst nematode (SBN) *H. schachtii* (pathotype 0 standard population MS) were incubated with the fungus. Healthy cysts from greenhouse propagated populations were surface-disinfected and placed either directly on or at the edge of fungal colonies. A sterility check was performed by imprinting the cysts in agar plates prior to inoculation. To examine the nematophagous ability of the fungus against nematode eggs, healthy looking nematode eggs were placed in the vicinity of fungal hyphae grown on PDA 1/3 using a modified version of Riddell’s slide culture technique (Gams et al. 1998). The methods applied here are described in detail in Ashrafi et al. (2017a).

#### Endophytic association studies towards wheat

Endophytic colonisation of wheat roots by the nematode-isolated strain DSM 106825 was examined using a modified axenic glass tube system (Kutter et al. 2006). To visualize hyphal colonization and potential changes in the root structure e.g. cell wall deposits associated with the ingress of hyphae, light microscopy techniques were applied including bright field (BF) and confocal laser scanning microscopy (CLSM).

The winter wheat cultivar Bezostaja was selected for the experiment, because it was the host cultivar of the cyst samples of *H*. *filipjevi*, from which the fungal strain DSM 106825 had been isolated. Seeds were surface disinfected with ethanol and sodium hypochlorite. Seeds were first washed with 1% Tween 20 (v/v) (Carl Roth, Germany) and shaken for 1 h at 120 rpm. The solution was discarded and seeds were incubated in 70% ethanol for 5 min followed by three rinses with SDW. Seeds were then washed in 5% sodium hypochlorite (Roth, Karlsruhe, Germany) for 30 min under shaking at 120 rpm, and rinsed six times with SDW. Seeds were incubated for 2 h in SDW and surface disinfected again with sodium hypochlorite for 10 min followed by six rinses with SDW. Seeds were air dried on sterilised filter papers under a laminar flow. Sterility was tested by imprinting the surface-disinfected seeds into Murashige & Skoog (MS) culture medium (Sigma-Aldrich, MO, USA). Seeds were then transferred to 2% water agar plates for germination. Plates were incubated at 25 °C in the dark until germination. Healthy looking seedlings showing no sign of visible contamination were used for the experiments.

Sterilised glass tubes (3 cm wide, 20 cm long) filled with washed and autoclaved quartz sand (3–5 mm) were used. Quartz particles were autoclaved at 134 °C for 15 min and dried at 100 °C overnight. Tubes were filled up to 10 cm with quartz particles covered with MS culture medium. One agar plug (0.5 mm diam.) of a fresh culture of strain DSM 106825 was placed on the MS medium and overlaid with 2 cm of autoclaved siliceous sand (1–1.5 mm). Tubes were sealed and incubated for 1 week at room condition to allow the fungus to grow and establish in the growth substrate. Then, a freshly germinated seed was transplanted into the glass tube and placed on the siliceous sand layer. Roots were covered with 1 cm of siliceous sand to hold the seedling in an upright position (Additional file 1: Fig. S1). Another sterilised glass tube was placed upside down on the main test tube and fixed with parafilm to extend the growing space for each plant. All glass tubes were incubated in the greenhouse at 20/16 °C day/night temperature and 16/8 light/dark cycle. Tubes with no fungal inoculum served as a control. The plants were sampled 8–10 weeks after incubation for microscopic observations, and to document potential alterations in the root system during the fungal colonization period. Prior to sampling, plants were visually examined for potential disease symptoms. Plants were gently removed from the growth substrates, and their roots were washed under running water to remove the remaining growth substrates.

Wheat roots were fixed and processed for staining following a modified protocol of Becker et al. (2018). Samples were incubated in 10% KOH for 3 h at room temperature and washed three times in phosphate-buffered saline (PBS; 137 mM NaCl, 2.68 mM KCl, 10 mM Na_2_HPO_4_, 1.76 mM KH_2_PO_4_, pH 7.4). The staining solution was prepared as follows: 10 µL of a 0.1% stock solution of Wheat Germ Agglutinin-Alexa Fluor™ 488 Conjugate (ThermoFisher Scientific, Massachusetts, USA), 20 µL of a 1% stock solution of Aniline Blue diammonium salt (C_37_H_32_N_5_O_9_S_3_) (Sigma-Aldrich, Germany), 10 µL of 2% Tween20 stock solution in 1 mL PBS 8, pH 7.4. Samples were stained by vacuum infiltration three times for 5 min in a glass desiccator (DWK Life Sciences). Samples were either transferred directly to microscopic slides or processed for longitudinal and transverse sectioning. Under a laminar flow, freshly stained roots were embedded into low melting agarose. A 5% agarose solution was prepared and root segments (3–5 cm) were imprinted into the cooling agarose gel poured into an embedding container. After solidifying, small blocks (1–2 cm) of agarose containing root fragments were cut off and sections of 100–150 µm thickness were prepared using a microtome (Microm GmbH, Walldorf, Germany).

Microscopic slides were prepared by mounting root fragments as well as the sections in a drop of water on microscopic slides, covered with a cover slips and examined by CLSM using a Leica TCS SP8 as described in Becker et al. (2018). Stained samples were excited with the Argon laser at 488 nm and the DPSS laser at 561 nm. Excitation at 488 nm was used to excite WGA-AF488 to visualize chitin, while excitation with 561 nm was used to excite the aniline blue fluorochrome to visualize glucan. Images were taken using the HC PLAPO CS2 20 × /0.75 IMM objective and the Application Suite X (LAS X; Leica) software for Leica microscopes.

For bright field microscopy, samples were observed and photographed by a Zeiss Axioskop 2 plus compound microscope equipped with a Jenoptik ProgRes® digital camera. Images were recorded using CapturePro 2.8 software.

## RESULTS

### Characterisation of fungal cultures

**Fig. 1:**
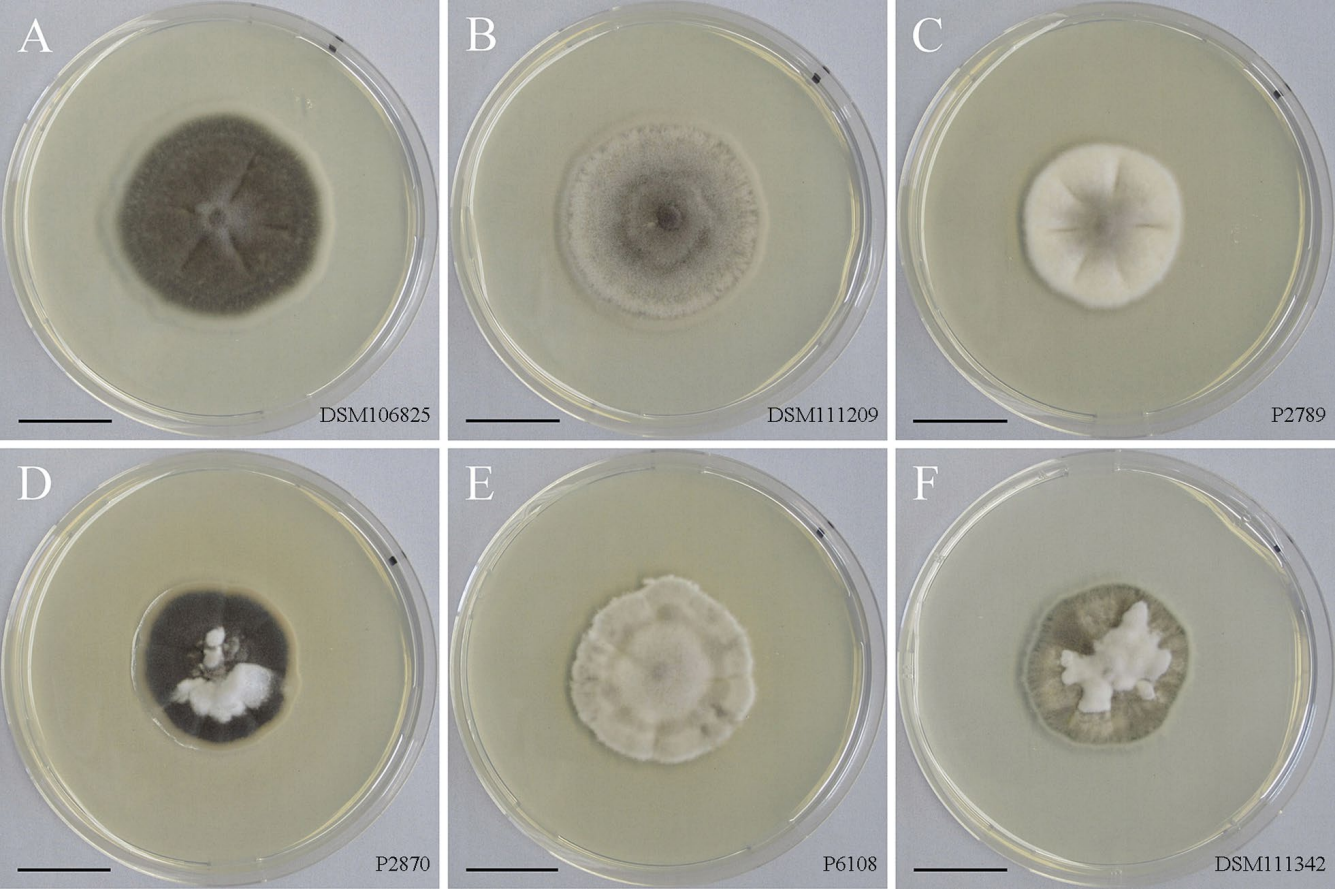
Colony morphology of the studied isolates grown on potato dextrose agar for 1 month. Strains number are given as inserts in the pictures. **a**, **b** strains DSM106825 (**a**) and DSM111209 (**b**) isolated from eggs of *Heterodera filipjevi*. **c**–**f** strains isolated from roots of *Microthlaspi perfoliatum*; strains P2789, 2870 and P6108 sampled from Germany, and strain DSM 111342 sampled from Bulgaria. Bars = 2 cm

Fungal strains isolated from nematode eggs and plant roots (Table 1), had similar growth characteristics, but differed in colony morphology and colour (Fig. 1). No conidia formation was observed in any of the strains, which were tested in different culture media under various growth conditions for up to 1 year.

The colony morphology of strain DSM106825 as the type strain of the fungal species studied here is reported from different culture media in more detail. On PDA the colony was slightly elevated centrally, velvet, olivaceous with a creamy greenish marginal zone on PDA (Fig. 1A). On MEA, the colony was flattened, centrally greenish grey, surrounded by a greenish black ring becoming greenish grey towards the margin. The CMA colony was flattened, greenish brown with sparse aerial mycelium. The fungus formed microsclerotia-like structures (Jumpponen and Trappe 1998; Knapp et al. 2012) on the surface of autoclaved wheat straw supplemented to SNA cultures and incubated at 20 °C for up to 3 months. These structures were aggregations of moniliform, thick-walled, and highly melanised hyphal cells (detailed in section: Taxonomy). Similarly, the microsclerotia-like structures were observed when the fungus grown on MMN agar plates was kept in water for up to 6 months to induce sporulation.

### Metabolite profiling

**Fig. 2:**
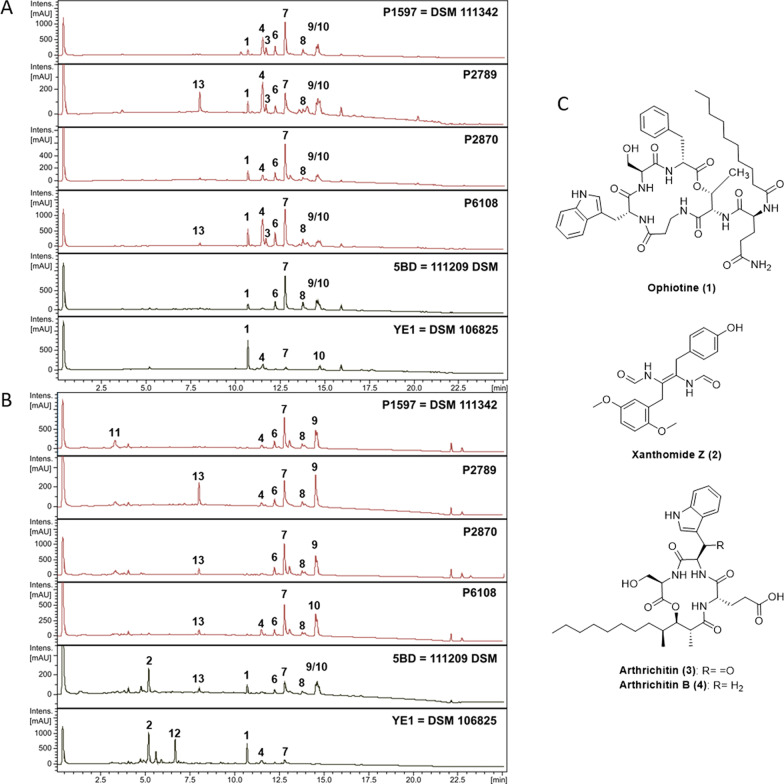
Comparison of HPLC–DAD/MS profiles of strains cultivated in ZM/2 media. Relative absorbance at 210 nm is shown. **a** Crude extracts from mycelia. **b** Crude extracts from supernatant. Crude extracts form plant-associated and nematode-associated strains are indicated in red and black, respectively. **c** Secondary metabolites (**1–4**) described by Helaly et al. (2018a) produced in ZM/2 media by the strain DSM 106825

In total six strains (Table 1) were cultivated in three different media and their crude extracts were analysed and compared for the occurrence of the major metabolites detected by HPLC–DAD/MS (Fig. 2, Additional file 1: Figs. S2 and S3). The metabolites ophiotine (**1**), xanthomide Z (**2**), arthrichitin (**3**) and artrichitin B previously described (Helaly et al. 2018a) were found in all isolates. In addition to these five already described compounds, seven other major peaks were included in the comparison. The results for all tested media are summarized in Additional file 1: Table S2. The results showed a high similarity between the HPLC–DAD/MS profiles of the studied isolates. Some metabolites were only produced by some or only one strain. Especially the peaks (**14**) and (**15**) were only produced by the plant isolated strains P2789, P2870 and P6108 (Additional file 1: Figs. S2 and S3). Futher investigation of these unkown compounds is ongoing.

### Phylogenetic inference

**Fig. 3:**
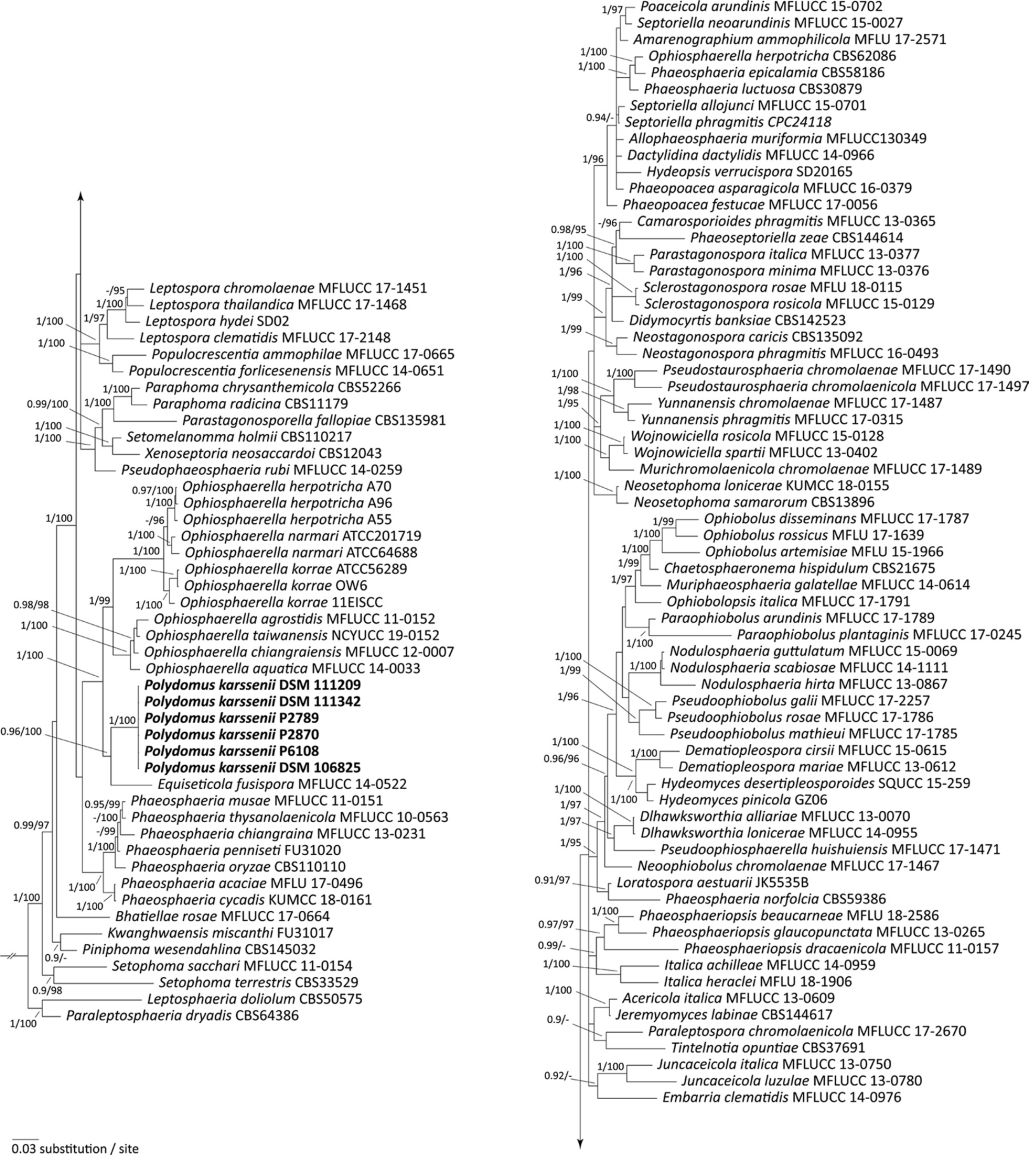
Bayesian inference of the phylogenetic relationship of the fungus described here among *Phaeosphaeriaceae* based on ITS, LSU, SSU, and *tefA* partial sequences using GTRI + I + G as the nucleotide substitution model. Depicted is a 50% majority rule consensus tree derived from 7500 trees from the stationary phase of a Monte Carlo Markov Chain. The process was run for 5,000,000 generation and trees were sampled every 500 generations. Numbers at the nodes are estimates of a posteriori probability (BIpp, ≥ 0.9) and ultrafast bootstrap values (UFBT ≥ 95%) given as BIpp/UFBT. The new species is highlighted in bold. The topology was rooted with the distantly related species *Leptosphaeria doliolum* and *Paraleptosphaeria dryadis*

The phylogenetic trees were based on concatenated alignments consisting of 3263 characters including gaps in the four-marker dataset (ITS: 545, LSU: 846, SSU: 982, tef1: 890) representing 112 strains and 99 taxa in the family of *Phaeosphaeriaceae* and *Leptosphaeria doliolum* and *Paraleptosphaeria dryadis* as the outgroup, and 4159 characters in the five-gene dataset (ITS: 588 characters, LSU: 835, SSU: 982 tef1: 880, rpb2: 874) representing 31 strains and 20 species. The different phylogenetic reconstruction methods (BI, ML, NJ) of the concatenated alignments resulted in highly similar tree topologies, and therefore only the BI tree is presented here (Fig. 3, Additional file 1: Fig. S4).

The DNA sequences obtained from the five genome regions were identical among all six strains of the here newly described species. The phylogenetic analyses accordingly clustered these strains, without any genetic distance among each other, as a distinct, highly supported monophyletic group in the four-gene (Fig. 3) and the five-gene trees (Additional file 1: Fig. S4). However, the sister group relationship of the new species differed between the four- and the five-gene trees: In the four-gene tree (Fig. 3) the strains formed a monophyletic group together with the monotypic genus *Equiseticola* and together they were sister to representatives of *Ophiosphaerella*, which consisted of two larger monophyletic subgroupings. The five-gene phylogeny also supported a monophyletic origin of the new genus with *Equiseticola* and *Ophiosphaerella*, but here the new species clustered as sister group to one of the two larger subgroups of *Ophiosphaerella* representing the species *O*. *herpotricha*, *O*. *korrae* and *O*. *narmari*, the causal agents of spring dead spot of Bermuda grass (Flores et al. 2017). *Equiseticola fusispora* clustered as sister to these, and a second group of *Ophiosphaerella*, consisting of Asian *Ophiosphaerella* species (Flores et al. 2017) including *O*. *agrostidis*, *O*. *taiwanenis*, *O*. *chiangraiensis*, *O*. *aquatica*, and *O*. *taiwanica* was sister to all of them rendering *Ophiosphaerella* paraphyletic (Additional file 1: Fig. S4).

### Pathogenicity against nematodes

**Fig. 4:**
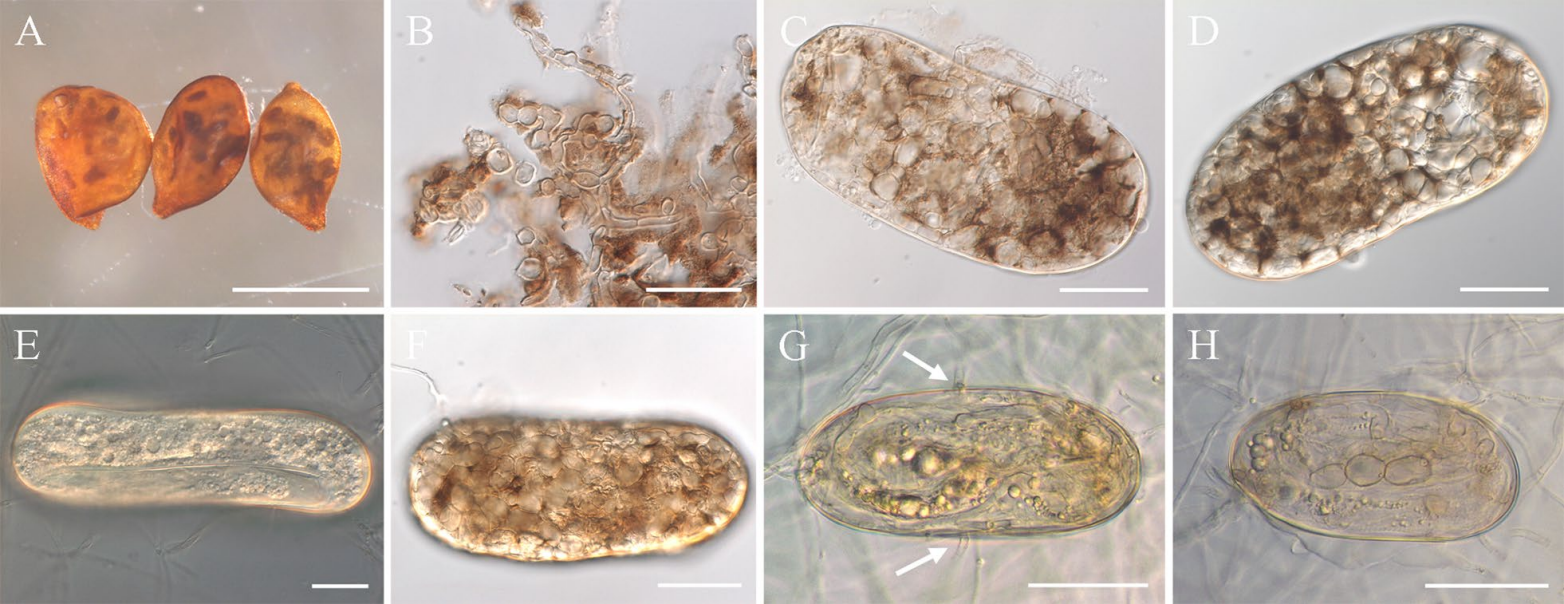
Pathogenicity of *Polydomus karssenii* DSM 106825 towards nematode cysts and eggs. **a** Symptomatic cysts of *Heterodera filipjevi* obtained from in vitro pathogenicity tests, exhibiting black eggs infected by the fungus. **b** Highly melanised running hyphae of *Polydomus karssenii* growing in cyst mucilage within the cysts cavity. **c–e** Fungal-infected eggs of *Heterodera filipjevi*: **c** Naturally occurring egg from field collected cyst samples infested by *Polydomus karssenii* (image from Helaly et al. (2018a), **d** Infested egg obtained from a cyst infected by the fungus during incubation on the fungal colony during in vitro tests, **e** Early stage of fungal colonisation observed in slide culture studies. Note deformation of the body cavity of the developing juvenile. **f–h** Fungal infected eggs of *Heterodera schachtii*: **f** Fungal infected egg obtained from a symptomatic cyst incubated on the fungus during in vitro pathogenicity tests; **g**, **h** In vitro infection process of *Polydomus karssenii* in nematode eggs: direct penetration of the fungus through the egg shell (indicated by arrows) and growing inside the body cavity of the developing juvenile (**g**), Development of the fungus inside the nematode body forming thick-walled moniliform hyphal cells (**h**). Bars = 0.6 mm (**a**), 30 µm (**b**–**d**, **f**), 20 µm (**e**), 50 µm (**g**, **h**)

In vitro pathogenicity tests were conducted using the strain DSM 106825. This strain was originally isolated from the eggs of *H*. *filipjevi*, and the pathogenicity tests showed that the fungus could parasitise cysts and eggs of *H. filipjevi *in vitro, and then be re-isolated in pure culture from this source, thus fulfilling Koch’s postulates. The cultures obtained were characterised based on morphology and DNA sequence comparisons, and were identical to original strain. The experiments additionally revealed that this strain could also parasitise cysts and eggs of the sugar beet cyst nematode (*H. schachtii*). In both cases initial indications of infection were observed in nematode cysts placed on the fungal colonies. The cysts were colonised by the fungus within 4–6 weeks (Fig. 4A). The colonised cysts resembled naturally diseased cysts as reported previously (Helaly et al. 2018a). Based on microscopic observations, the cyst colonisation started by fungal growth into the cyst mucilage in the cyst cavity, subsequently followed by fungal ingress via the eggshell and development inside the body cavities of developing juveniles (juveniles inside the eggs). The ‘runner hyphae’ growing into the cyst mucilage were moniliform and strongly melanised (Fig. 4B) as well as the developing hyphae colonising the eggs. The body cavity of developing juveniles were entirely colonised by the fungus resulting in dark brown to black discolouration of the infested eggs (Fig. 4C, D).

Slide culture experiments, where only individual eggs were placed in some distance to the fungus showed that the fungus penetrated the shell of the eggs directly. No formation of specialised infection structures e.g*.* appressoria were observed. Following fungal ingress into the eggs, the hyphae penetrated the body cuticle of developing juveniles and colonised the nematode body cavity by developing enlarged and moniliform cells (Fig. 4E–H).

### Endophytic colonisation of wheat roots

The ability of strain DSM 106825 to endophytically colonise roots of wheat plants was examined in vitro. Plants inoculated with the fungus had well-developed root systems, similar to those in the uninoculated controls. No development of disease symptoms were observed on root or shoot tissues.

**Fig. 5:**
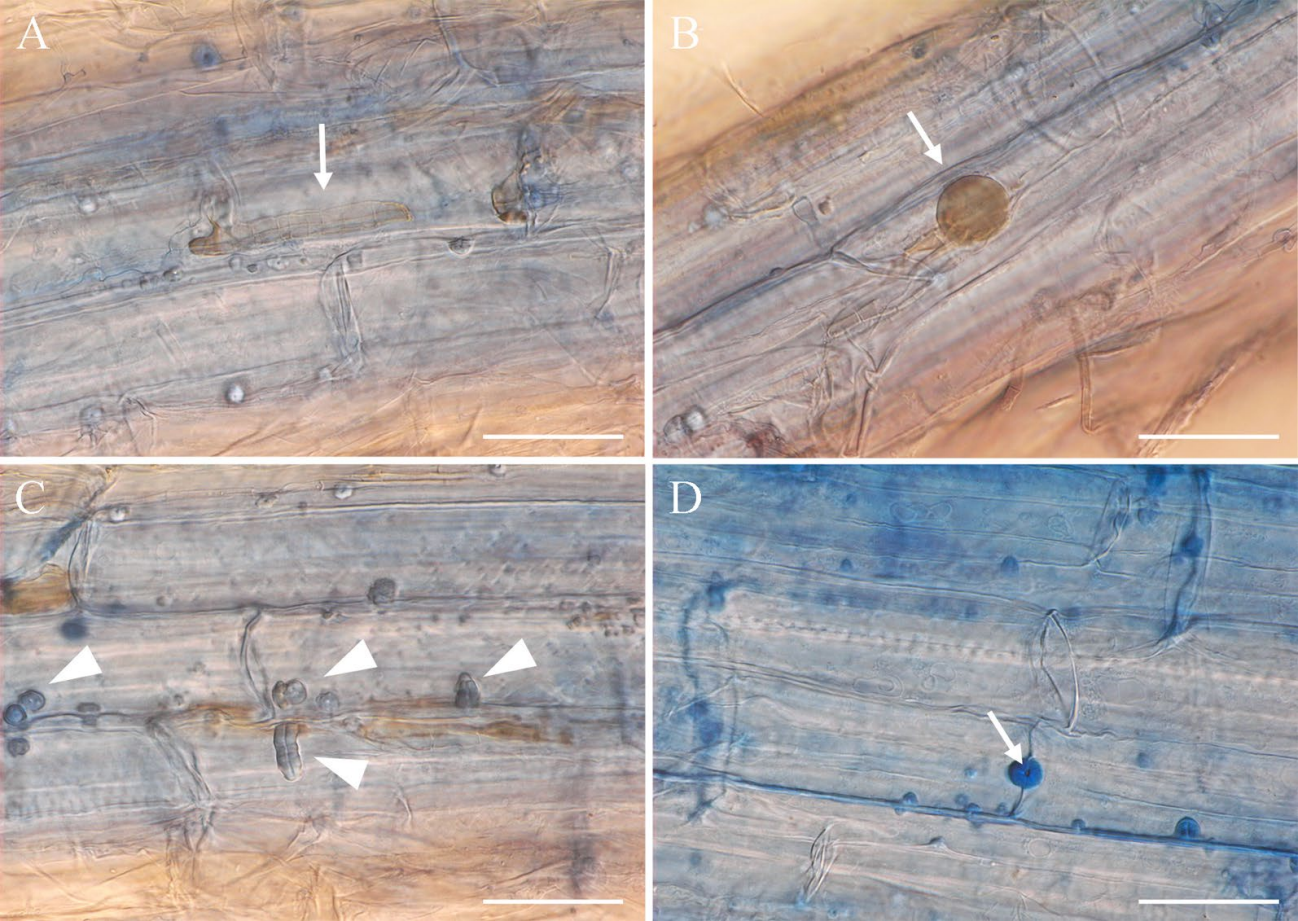
Light micrographs of interaction between *Polydomus karssenii* and wheat roots. **a**, **b** Formation of microsclerotia (arrows) inside the plant cells. **c** Formation of callus tissues/papillae (arrowheads) assumingly as a plant response to fungal colonisation. **d** *En face* view of a callosic papilla-like structure formed between two adjacent root cells; the sample was stained with cotton blue. Plants grown on MS medium. Bars = 30 µm

Bright-field microscopy revealed that the fungus formed melanised hyphae, intracellular microsclerotia and chlamydospore-like structures in plant roots (Fig. 5A, B). These features were consistent with the definition of dark septate endophytes, and for example with those reported by Knapp et al. (2012, 2015), who investigated the interaction of various DSEs with their host plants in vitro.

**Fig. 6:**
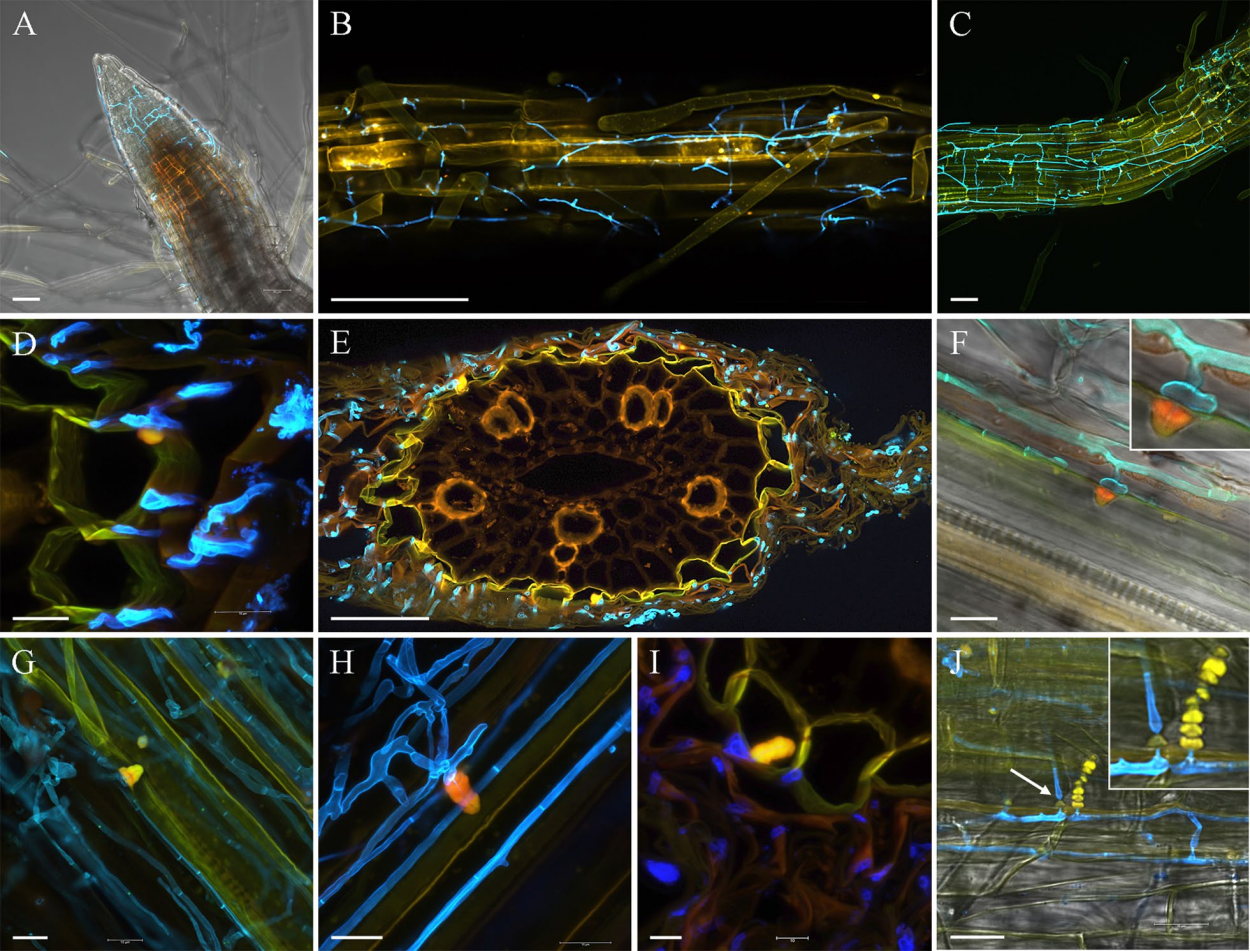
Confocal laser scanning micrographs of fungus–plant cell interaction between *Polydomus karssenii* strain DSM 106825 and wheat. Wheat seedlings (1 week old) were inoculated with mycelium plugs and infection monitored 8–9 weeks thereafter. Infected plant roots were treated with aniline blue diammonium salt to stain fungal and plant cell wall “β-glucans" (overlay of stain and autofluorescence of cytoplasm depicted in yellow, or orange pseudo colours, respectively) and wheat germ agglutinin-Alexa Fluor 488 (WGA-AF488) to stain fungal chitin (blue/turquoise pseudo colour). **a**–**c** General view of root apex showing fungal colonisation. **d** Transverse section of root showing intercellular colonisation of root cells by strain DSM 106825. **e** Transverse section of vascular cylinder (stele) showing that the fungus colonised cortex and endodermal cells but not the vascular tissues. **f** Longitudinal section showing hyphal swelling, resembling an appressorium, and formation of a penetration peg surrounded by a callosic papilla-like structure. **g**, **h** Longitudinal section showing the development of callosic papilla-like structures surrounding hyphal penetration pegs. **i** Transverse section showing intercellular colonisation of the fungus and formation of callostic papilla-like structures associated with the fungal colonisation of plant cells. **j** Longitudinal section indicating the growth of a hypha by passing through callosic tissues (papillae like structures). Bars = 50 µm (**a–c**), 10 µm (**d–j**)

Anilline blue and WGA-AF488 stained samples were processed for confocal microscopy for a detailed study of root colonisation by strain DSM 106825. The fungus developed on the root surface covering the apical meristem and upper parts of the roots (Fig. 6A–C). The fungus colonised epidermal, cortex and endodermal cells growing intercellular (Fig. 6D), but was not observed in the vascular cylinder (Fig. 6E). The intercellular hyphae regularly formed swellings, resembling appressoria, from which minute, elongated hyphal peg-like structures developed upon contacting the plant cell wall (Fig. 6F). As shown in Fig. 6F–J, however, these peg-like structures were entirely ensheathed by callosic papilla-like structures. The photographs show the presence of a minor opening along the callosic papilla-like structures providing a canal-like path for cell wall intrusion (Figs. 5C, D; Fig. 6F–I). The canal was clearly discernible, via which the peg-like hyphae passed through the plant cell wall and successively entered the plant cell (Fig. 6F–I). The callosic papilla-like tissues extended into plant cells surrounding the hyphal pegs (Fig. 6I). It was occasionally observed that fungal hyphae continued growing after passing through the callose tissues and returned into the usual size and form (Fig. 6J). This event was not observed frequently making it difficult to clarify the further steps of mycelial growth.

## Taxonomy

***Polydomus*** S. Ashrafi & W. Maier gen. nov.

MycoBank No. MB846360.

*Etymology***:** Poly (Greek), indicating more than one, much and many, and domus (Latin) meaning homes or habitats.

*Typification: Polydomus karssenii* S. Ashrafi**,** J. G. Maciá-Vicente & W. Maier.

*Diagnostics***:** Colony morphology including shape, growth characteristics and the absence of sexual spores is similar among the strains. No conidia were observed. Colonies varied in colour ranging from olivaceous to pale creamy green, radially striate. Strains were isolated from surface-disinfected eggs of the cereal cyst nematode *Heterodera filipjevi* collected from Turkey, or surface-disinfected roots of the *Brassicaceae* species *Microthlaspi perfoliatum* collected from Germany and Bulgaria. These fungal isolates did not produce fruiting bodies or spores of any kind under a variety of cultural conditions.

*Discussion: Polydomus* currently is a monotypic genus, phylogenetically forming a highly-supported sister relationship with the monotypic genus *Equiseticola*, and a monophyletic lineage of the North American and Australian isolates of *Ophiosphaerella* species (Flores et al. 2017) including *O. herpotricha*, *O. korrae*, and *O. narmari*.

***Polydomus karssenii*** S. Ashrafi**,** J. G. Maciá-Vicente & W. Maier **sp. nov.**

MycoBank No. MB 846361.

Figure 7

**Fig. 7:**
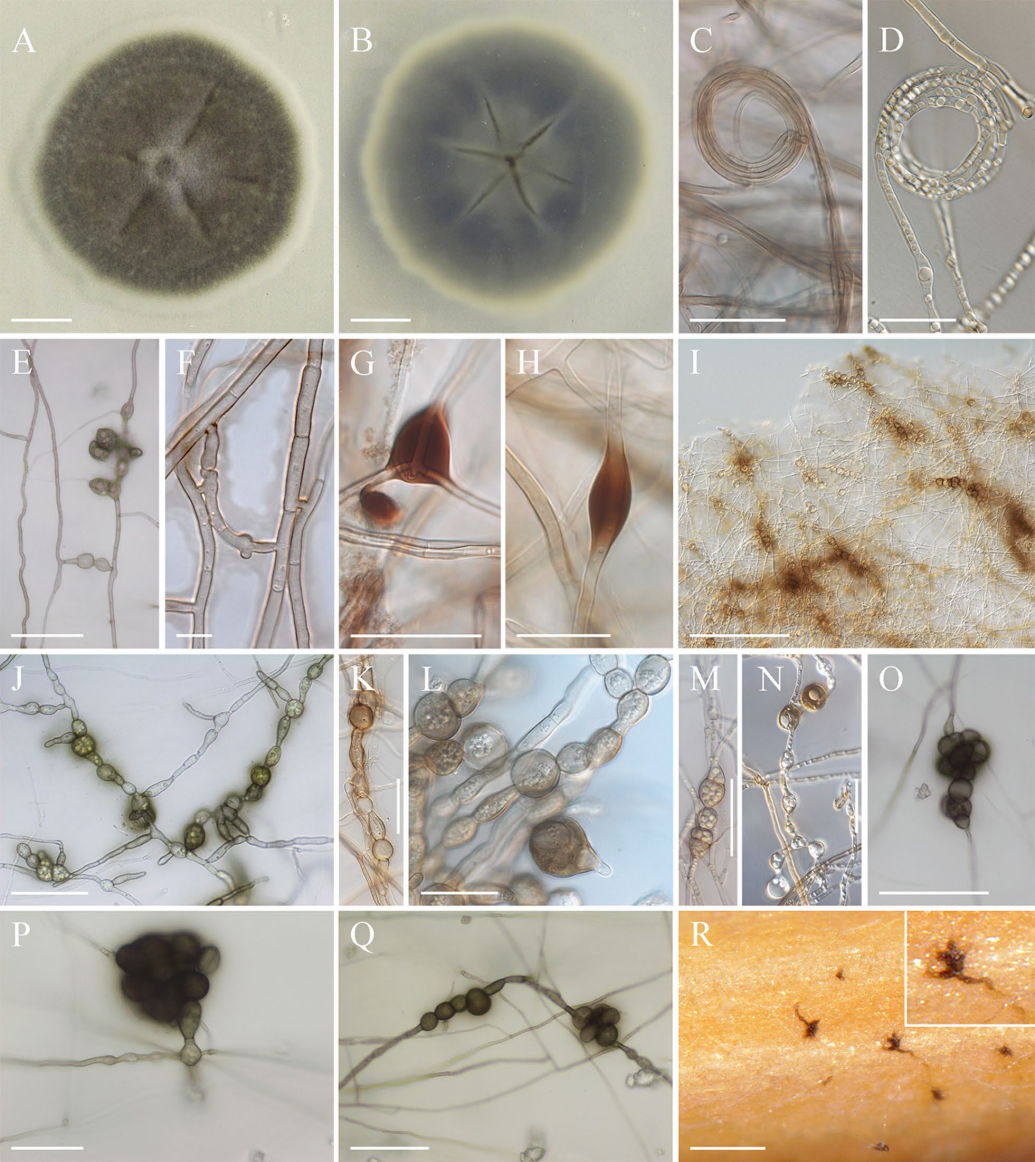
Micrographs of *Polydomus karssenii*. **a**, **b** Isolate growing on PDA, top view (**a**) and bottom view (**b**). **c**, **d** Fungal growth and formation of coiling hyphae. **e**, **f** Hyphal anastomosis. **g**, **h** Formation of pigmented hyphal vesicle-like structures. **i**–**n** Fungal growth and formation of moniliform hyphal cells (**j**, **k**) and chlamydospore-like structures (**l**–**n**). **o**–**r** Formation of highly melanised microsclerotia structures, in culture media (**o**–**q**), on wheat straw (**r**). A, B, C, F: from PDA; I, K, L, M: from PDA1/3; J: from PDA 1/6; E, O, P, Q: from CaCO_3_; G, H: from CZA; N: from MEA 1%. Bars = 1 cm (**a**, **b**), 200 µm (**i**, **r**), 50 µm (**e**, **j**, **m**, **o**, **q**), 30 µm (**c**, **g**, **k**, **n**, **p**), 20 µm (**d**, **h**, **l**), 10 µm (**f**)

*Etymology*: In honour of Gerrit Karssen for his outstanding contributions to the field of nematology, especially in expanding our knowledge on plant parasitic nematodes.

*Diagnosis:* Naturally occurring infected nematode eggs displaying brownish discolouration, colonised by inflated, moniliform and melanised hyphae in body cavities of developing nematode juveniles.

*Type:* Turkey, Yozgat, a dried biologically inert culture on PDA, originating from an individual nematode egg from a cyst of *Heterodera filipjevi*, isolated by Samad Ashrafi, August 2013, dried culture on PDA, deposited at the herbarium of the Botanic Garden and Botanical Museum Berlin-Dahlem: B 70 0100748.

*Ex-type culture:* DSM 106825 (YE1), preserved in a metabolically inactive state at the open collection of the Leibniz-Institut DSMZ (German Collection of Microorganisms and Cell Cultures GmbH). GenBank accession numbers of isotype sequences: ITS: ON407111; LSU: ON407074; SSU: ON408345; *rpb2*: ON419510; *tef*A: ON419499.

*Additional material examined:* Turkey, Yozgat, DSM 111209 (5BD), isolated from a single nematode egg from a cyst of the cereal cyst nematode *Heterodera filipjevi*, August 2013, Samad Ashrafi, GenBank accession number: ON407112 (ITS), ON407075 (LSU), ON408346 (SSU), ON419511 (*rpb*2), ON419500 (*tef*A). Bulgaria, Diviya, DSM111342 (P1597) obtained from surface sterilised roots of *Microthlaspi perfoliatum*, May 2013, K. Glynou & J.G. Maciá-Vicente, GenBank accession number: ON407113 (ITS), ON407076 (LSU), ON408347 (SSU), ON419512 (*rpb*2), ON419501 (*tef*A). Germany, P2789, in the roots of *Microthlaspi perfoliatum*, May 2013, K. Glynou & J.G. Maciá-Vicente, GenBank accession number: ON407114 (ITS), ON407077 (LSU), ON408348 (SSU), ON419513 (*rpb*2), ON419502 (*tef*A); ibid, P2870, GenBank accession number: ON407115 (ITS), ON407078 (LSU), ON408349 (SSU), ON419514 (*rpb*2), ON419503 (*tef*A); ibid, P6108, GenBank accession number: ON407116 (ITS), ON407079 (LSU), ON408350 (SSU), ON419515 (*rpb*2), ON419504 (*tef*A).

*Description:* Colonies moderate in growth, at 20 °C on PDA reaching 8–10 mm diam. (7 days), 17–20 mm (14 days), 26–30 (21 days); optimum temperature for growth 20, no growth observed at 35 °C; optimum temperature for growth in other examined cultural media 20 °C, after 21 days reaching 37–41 mm diam. (CMA), 56–58 mm (CZ), 39–41 mm (MEA), 42–45 mm (MMN), 58–62 mm (MS), 66–71 mm (OA), 53–56 mm (SNA).

Colonies on PDA slightly elevated centrally, radially striate; colony surface velvet, brownish‐grey to olivaceous brown (Bark green RAL 090 30 20 to olive black RAL 080 30 10) towards the margin, margins pale creamy, no medium staining; Colony reverse pale olivaceous in centre to greenish grey towards the margin, margin pale creamy. Hyphae hyaline or highly melanised, septate, thick walled, forming coils and anastomosis, on CaCO_3_, CZA, MEA 1%, OA and SNA, forming occasionally elongated and highly melanised vesicles-like structures, hyphal cells occasionally globose to subglobose, moniliform as colonies age. Conidiophores and conidia not observed. Chlamydospore or dictyochlamydospore-like structures occasionally developing intercalary or terminally at the side branches of 6–8 week-old PDA1/3, CaCO_3_, and MEA1% agar plates, filled with small guttules, gradually pigmented, interweaving to form highly pigmented microsclerotia-like structures. Microsclerotial structures were formed also on the surface of autoclaved wheat straw supplemented to SNA cultures. Sexual state (Teleomorph) not observed.

*Discussion:* Strains of *Polydomus karssenii* associate either with plant parasitic nematodes as egg-parasitic fungus, or with plant roots as endophytes. According to phylogenetic inferences (Fig. 3, Additional file 1: Figs. S4, S5) *Polydomus karssenii* has a close affinity with, but distinct from the representatives of *Ophiosphaerella* and the monotypic genus *Equiseticola*. The sexual morph of *Polydomus karssenii* was not found. This is in accordance with other DSEs reported previously (Ashrafi et al. 2018; Grünig et al. 2008; Knapp et al. 2012; Romero-Jiménez et al. 2022; Yuan et al. 2020b). On the other hand an asexual morph has not been reported for the species of *Ophiosphaerella* or *Equiseticola,* except for *O. agrostidis* (Thambugala et al. 2017)*,* which produces conidiomata and conidia. These structures, however, were not observed in *Polydomus karssenii*. This situation makes the morphological comparison between the fungus and its closely related species difficult. Nevertheless, production of chlamydospore-like and vesicle-like structures, hyphal coils and anastomoses, and lack of conidia formation are the morphological characteristics of *Polydomus karssenii*, which have not been reported from its closely related species. Ecologically, *Polydomus karssenii* can be separated from other fungi discussed here by its host range and lifestyle. While species of *Ophiosphaerella* and *E*. *fusispora* are pathogenic or saprobic (Abd-Elsalam et al. 2016; Flores et al. 2017), *Polydomus karssenii* has a bifunctional lifestyle, parasitising eggs of plant parasitic nematodes on the one hand and living as a root endophyte of *Poaceae* and *Brassicaceae* on the other hand.

## DISCUSSION

### Taxonomic relations

In this study the monotypic genus *Polydomus* was described based on phylogenetic inferences, morphological characteristics and the lifestyle of the fungus. The genus *Polydomus* was established to accommodate the plant and nematode associated novel species *Polydomus karssenii*, strains of which were isolated either from the eggs of the CCN *H*. *filipjevi* or the roots of the brassicaceous plant *M. perfoliatum*. Phylogenetic inference placed the studied representatives of *Polydomus* as a highly supported monophyletic group into the *Phaeosphaeriaceae* (Barr 1979), one of the most species-rich families in *Dothideomycetes* with 126 genera listed in MycoBank (https://www.mycobank.org/page/Simple%20names%20search). The representatives of *Phaeosphaeriaceae* have been reported from a wide variety of terrestrial and aquatic habitats (Phookamsak et al. 2014; Tennakoon et al. 2020). The family comprises saprobic, endophytic and plant pathogenic representatives, especially on monocotyledonous plants (Phookamsak et al. 2014; Tennakoon et al. 2020), as well as the recently introduced fungi *Tintelnotia* spp. as human pathogens (Ahmed et al. 2017). Morphologically, they are mainly characterised by immersed to erumpent and superficial, (sub-)globose ascomata, short papilla and bitunicate asci (Barr 1979; Tennakoon et al. 2020). To our knowledge however there has been no report on any phaeosphaeriaceous species inhabiting the nematode cyst cavities, and parasitising eggs of plant parasitic cyst nematodes so far.

*Polydomus* clustered as sister group to the also monotypic genus *Equiseticola*, which had been based on *E. fusispora* isolated from the stems of horsetail plants (*Equisetum* sp.) in Italy. This genus clearly differs from *Ophiosphaerella*, not only phylogenetically, but also relating to the morphology of peridia, pedicels as well as ascospores (compare Abd-Elsalam et al. (2016)). Several *Phaeosphaeria* species (*Phaeosphaeria arenaria, P. epicalamia, P. huronensis, P. luctuosa, P. nofolcia, P. recessa* and *P. saronica*) however, have also similar, 5-septate ascospores as was mentioned for *Equiseticola* by Abd-Elsalam et al. (2016). Therefore, an effort was made to include some of these species that had not been studied phylogenetically before into the analyses. We obtained 3 strains from these species and sequenced them for all markers. The sequences of *P. epicalamia* and *P. luctuosa* grouped highly supported together with one strain of *O. herpotricha* (CBS620.86). It is interesting to note, but any consequences outside of the scope of this paper, that the strain *O. herpotricha* (CBS620.86) reported from Switzerland (Walker 1980) did not group with the other three sampled strains of that species, which clustered within the highly supported *Ophiosphaerella* sub-clade comprising all three species causing spring dead spot of Bermuda grass (*i.e. O*. *herpotricha*, *O*. *korrae* and *O*. *narmari*). Additionally, the two *Phaeosphaeria* spp. did not cluster with the main *Phaeosphaeria* group in all phylogenetic trees (Fig. 3, Additional file 1: S4). The placement of this monophyletic group comprising of *P. epicalamia, P. luctuosa* and *O. herpotricha* (CBS620.86) within *Phaeosphaeriaceae* thus remained inconclusive suggesting for all three specimens that they potentially should be relegated to another genus after more detailed analyses. All other representatives of *Ophiosphaerella* grouped monophyletically in the four-marker tree (Fig. 3).

In the five-marker tree with the much smaller taxon sampling (Additional file 1: Fig. S4), *Ophiosphaerella* was rendered paraphyletic by *Polydomus* and *Equiseticola* that both grouped as individual monophyla between the two subgroups of *Ophiosphaerella* (compare results). The phylogenetic analysis highly supported a sister group relationship between *Polydomus karssenii* and a monophyletic clade of *Ophiosphaerella* consisting of the species *O*. *herpotricha*, *O*. *korrae*, and *O*. *narmari* (Additional file 1: Fig. S4). These three species are the causal agents of spring dead spot of Bermuda grass from North America and Australia (Flores et al. 2017). the other group of *Ophiosphaerella*, here referred to as the South Asian lineage, including *O. agrostidis*, *O. aquatica*, *O. chiangraiensis*, *O. taiwanica*, and *O. taiwanensis* (Ariyawansa et al. 2015; Ariyawansa and Jones 2019; Camara et al. 2000; Tennakoon et al. 2020; Yuan et al. 2020a) formed a separate clade clustering with North American and Australian *Ophiosphaerella* species, *Polydomus karssenii* and *Equiseticola*. To further elucidate the placement of the here described genus *Polydomus,* as well as that of its close relative *Equiseticola* within *Phaeosphaeriaceae*, it will be important to especially sample more species within *Ophiosphaerella* including the type species, *O. graminicola* originating from Argentina (Spegazzini 1909), and also more loci. Taken together, the here described monotypic genus *Polydomus* differs clearly from *Equiseticola* and from *Ophiosphaerella* by a highly supported monophyletic placement within the phylogenetic trees, the absence of any sexual structures and its life style as root endophyte and nematode egg parasite.

The phylogeny of *Polydomus karssenii* suggests that nematode parasitising strains are conspecific with the endophytic strains, since both groups of isolates showed identical DNA sequences. Other than minor intraspecific variabilities in colony morphology, all strains showed similar growth characteristics and had similar metabolite profiles. Both nematode and plant isolated strains could produce the major compounds previously described from the nematode isolated strain DSM 106825 (Helaly et al. 2018a). Minor differences e.g*.* in colour of the colonies or in metabolite profiles among the strains could derive from different ecological factors. *Polydomus karssenii* was isolated from plant and cyst nematode species that are geographically distributed from the Western to the Eastern Europe and Asia Minor. Differences in climate, latitude, light and UV irradiation (which can induce pigmentation), as well as in the lifestyle and host interaction of the fungus, could induce phenotypic alterations among its strains (Bazzicalupo 2022).

### *Polydomus karssenii* as endophyte

Since the nematode-isolated strain of *Polydomus karssenii* was initially isolated from a wheat parasitising cyst nematode, this prompted us to use the same strain to study its ability in endophytic colonisation of wheat roots, while other strains had already been isolated from *M*. *perfoliatum* as endophytes. Microscopic observations showed a colonisation pattern similar to the endophytic and nematode egg parasitic fungus *Pochonia chlamydosporia* on barley and tomato roots (Escudero and Lopez-Llorca 2012; Maciá-Vicente et al. 2009). Both fungi can colonise root cells but not the root vascular cylinder, develop appressoria-like structures, and induce formation of papilla-like structures. However, *Polydomus karssenii* develops predominantly intercellular hyphal growth. A most parsimonious explanation for formation of the papilla-like structures is a plant response to microbial intrusion (Voigt 2014). The plant cell wall, as the first physical defence barrier, could be supported by formation of cell wall appositions such as papillae at the site of microbial invasion (Underwood 2012; Voigt 2014), covering or halting the infection structures (Honegger 1986; Underwood 2012; Voigt 2014). This phenomenon occurs among different fungi ranging from lichen-forming (Honegger 1986) to pathogenic (Underwood 2012) species, as well as among endophytes (Maciá-Vicente et al. 2009). However, Currah et al. (1993) studied morphological alterations in *Rhododendron brachycarpum* colonised by *Phialocephala fortinii* as one of the well-studied DSEs, and reported that root colonisation resulted only in deposition of a collar of cell wall material around the entering hyphae (Peterson et al. 2008). It can also be speculated that formation of callose might act as shield protecting the fungal mycelium from recognition and degradation while intruding plant cells. This can be related to endophytic behaviour of the fungus and be interpreted as a strategy to suppress  the plant immune system during the colonisation process.

The microscopic observations suggest that *Polydomus karssenii* is a dark septate endophyte with a multifunctional lifestyle and it can be defined as a nematode antagonistic endophyte. It endophytically colonised both wheat (present study) and *M*. *perfoliatum* (Glynou et al. 2016) as monocotyledonous and dicotyledonous plants, respectively. The fungus has frequently been reported colonising plants in arable soils. There are several ITS fungal sequences available on GenBank, which are identical or highly similar (> 99.5%) to those of *Polydomus karssenii*. These sequences were reported in different ecological studies and obtained mostly from cereal growing systems such as maize, wheat, and oat in different European countries including Germany (Moll et al. 2016), France (Comby et al. 2016), Sweden (Grudzinska-Sterno et al. 2016), and the UK (Carter et al. 1999). The wide distribution, occurrence in various ecosystems, and endophytic association with mono- and dicots suggest *Polydomus karssenii* as a generalist endophyte.

### *Polydomus karssenii* as nematode egg parasite

The fungal infection of nematode eggs observed in this study resembled the infection process in the previously studied DSEs *P. sieberi* (Ashrafi et al. 2018) and a recently described *Laburnicola* species, *L*. *nematophila* (Knapp et al. 2022). Despite formation of appressoria-like structures during plant colonisation, these infection structures were not observed when the fungus colonised the nematode eggs. It suggests that endophytic behaviour and the lifestyle of the fungus could have a determining influence on the fungal morphology, i.e*.* appressorium initiation might be required as a part of plant colonisation strategy. In any case, either nematode egg or plant cell, we did not observe cell wall distortion at the site of penetration, suggesting that in addition to physical pressure an enzymatic or chemical mechanism might be involved in the colonisation process. Based on our microscopic observation, *Polydomus karssenii* parasitised the eggs of *H. filipjevi* and *H. schachtii* using hyphal penetration as the general mode of action for nematode egg-parasitic fungi (Nordbring-Hertz et al. 2011). In this group of fungi, the hyphal ingress into nematode eggs continues by penetration of the body cuticle of the developing juvenile inside the egg cavity, and digests the developing juvenile. We have previously reported that *Polydomus karssenii* produces various bioactive compounds, among which ophiotine showed a nematicidal effect (Helaly et al. 2018a). In addition to direct hyphal colonisation, we therefore assume that the fungus employs production of bioactive compounds, in particular ophiotine, to colonise nematode eggs. It seems likely that by producing nematicidal compounds, the fungus can kill or negatively affect the viability and vitality of the developing juveniles, which are later colonised using hyphal penetration as a physical colonisation mechanism.

*Polydomus karssenii* was capable of colonising the nematode cyst cavities. Cyst structures are nutrient-rich and protective micro-environments, where the nematode eggs can survive for many years in absence of host plants (Jones et al. 2013). According to the lifestyle of the fungus being able to colonise both plant roots and nematode eggs and cysts, it can be speculated that the fungus can exploit the nematode cysts and their egg content as another strategy for further development and survival, especially in the absence of the host plant.

### Chemotaxonomic relevance of the secondary metabolites

*Polydomus karssenii* produces a set of secondary metabolites with various biological activities including nematicidal effects (Helaly et al. 2018a). These results are in accordance with previous studies where the secondary metabolite profiles were found to be specific for a certain fungal taxon (cf. Frisvad et al. 2008; Helaly et al. 2018b for an overview). All nematode and plant-isolated strains could produce the previously identified major compounds, among others the nematicidal compound ophiotine (Helaly et al. 2018a). A preliminary study on similar aspects was already carried out on another taxonomic group of nematode-associated organisms and also revealed convergent secondary metabolite profiles in the clavicipitaceous genus *Pochonia* (Stadler et al. 2003). The various yet unknown metabolites that were detected in the extracts as minor metabolites will now be isolated, identified and subjected to studies on their biological activities. This will finally allow for conclusions as to their ecological role and their chemotaxonomic significance.

## CONCLUSION

In this study the new genus *Polydomus* was erected to accommodate the new species *P. karssenii*. The fungus is a dark septate endophytic and nematode-parasitic fungus, which was isolated from eggs of the CCN *H. filipjevi*, and the plant species *M. perfoliatum*. *Polydomus karssenii* produces a set of secondary metabolites with different bioactivity ranging from nematicidal to antifungal effects. The fungus colonises the host plant by developing intercellular hyphae. Within the root tissues it forms appressorium-like structures starting an intriguing interaction with adjacent plant cells that needs to be further investigated to be properly understood. To parasitise nematode eggs, *P. karssenii* penetrates the eggshells and cuticle of developing juveniles, and colonises their body cavities. The morphologically intraspecific variabilities among the fungal strains, broad geographical distribution, inter-kingdom host interactions (i.e. nematode parasitism—plant endophytism) suggest *P. karssenii* as an ecologically multifunctional DSE.

## Supplementary Information

**Additional file 1.** Experimental procedures, comparison of HPLC-DAD/MS profiles, additional phylogenetic details of the fungus.

## Data Availability

All sequences generated during this study have been submitted to GenBank. Alignments and phylogenetic trees have been submitted to Figshare repository (https://figshare.com/) and can be accessed via this link: 10.6084/m9.figshare.21558846.

## Abbreviations

AIC: Akaike information criterion
BI: Bayesian inference
BF: Bright field microscopy
CLSM: Confocal laser scanning microscopy
CCN: Cereal cyst nematode
CMA: Cornmeal agar
CNs: Cyst forming nematodes
CTAB: Cetyl trimethylammonium bromide
CZA: Czapek Dox agar
DSE: Dark septate endophytes
GTR + I + G: General time reversible model with gamma distributed substitution rates and invariate sites
hLRT: Hierarchical likelihood ratio test
HPLC–DAD/MS: High-performance liquid chromatography coupled with diode-array detection and electrospray ionization tandem mass spectrometry
ITS: Internal transcribed spacers
LSU: Large subunit of the ribosomal RNA
MCMC: Markov Chain Monte Carlo
MEA: Malt extract agar
ML: Maximum-likelihood
MMN: Modified Melin-Norkrans medium
MS: Murashige and Skoog
OA: Oatmeal agar
PCR: Polymerase chain reaction
PDA: Potato dextrose agar
PPNs: Plant parasitic nematodes
RAxML: Randomized axelerated maximum likelihood
*rpb2*: RNA polymerase II second-largest subunit
SBN: Sugar beet cyst nematode
SDW: Sterilised deionised water
SNA: Synthetic nutrient-poor agar
SSU: Small subunit of the ribosomal RNA
tef1: Translation elongation factor 1-α
WGAAF488: Wheat germ agglutinin-Alexa Fluor 488
