# Supplementary material for: Polydomus karssenii gen. nov. sp. nov. is a dark septate endophyte with a bifunctional lifestyle parasitising eggs of plant parasitic cyst nematodes (Heterodera spp.)
Source: IMA Fungus. 2023 Mar 30;14:6. doi: 10.1186/s43008-023-00113-w (PMC10064538; doi:10.1186/s43008-023-00113-w)
Supplement: Supplementary file 1 — Additional file 1. Experimental procedures, comparison of HPLC-DAD/MS profiles, additional phylogenetic details of the fungus. [file 43008_2023_113_MOESM1_ESM.docx]

Supporting Information

*Polydomus karssenii* gen. nov. sp. nov. is a dark septate endophyte with a bifunctional lifestyle parasitising eggs of plant parasitic cyst nematodes (*Heterodera* spp.)

Samad Ashrafi^1, 2 *^, Jan-Peer Wennrich^3, 4^, Yvonne Becker^1^, Jose G. Macia-Vicente^5^, Anke Brißke-Rode^1^, Matthias Daub^6^, Torsten Thünen^2^, Abdelfattah A. Dababat^7^, Maria R. Finckh^8^, Marc Stadler^3, 4^, Wolfgang Maier^1^

^1^Institute for Epidemiology and Pathogen Diagnostics, Julius Kühn Institute (JKI) – Federal Research Centre for Cultivated Plants, Messeweg 11/12, 38104 Braunschweig, Germany.

^2^ Institute for Crop and Soil Science, Julius Kühn Institute (JKI) – Federal Research Centre for Cultivated Plants, Bundesallee 58, 38116 Braunschweig, Germany.

^3^ Department Microbial Drugs, Helmholtz Centre for Infection Research, Inhoffenstraße 7, 38124 Braunschweig, Germany.

^4^ Institute of Microbiology, Technische Universität Braunschweig, Spielmannstraße 7, 38106 Braunschweig, Germany.

^5^ Plant Ecology and Nature Conservation, Wageningen University & Research, PO Box 47, 6700 AA Wageningen, the Netherlands.

^6^ Institute for Plant Protection in Field Crops and Grassland, Julius Kühn Institute (JKI) – Federal Research Centre for Cultivated Plants, Dürener Str. 71, 50189, Elsdorf, Germany.

^7^ International Maize and Wheat Improvement Centre (CIMMYT), P.O. Box 39, Emek, 06511 Ankara, Türkiye.

^8^ Department of Ecological Plant Protection, University of Kassel, Witzenhausen, Germany.

*Correspondence: [samad.ashrafi@julius-kuehn.de](mailto:samad.ashrafi@julius-kuehn.de)

## Contents

**Figure S1.** Schematic overview of the axenic glass tube system to demonstrate fungal colonization process and potential structural changes in root cells. Tube were filled by quartz and MS culture medium. 3

**Figure S2.** Comparison of HPLC-DAD/MS profiles of strains cultivated in Q6/2 media. Relative absorbance at 210 nm is shown. A) crude extracts from mycelia, B) crude extracts from supernatant. Crude extracts form plant-associated and nematode‑associated strains are indicated in red and black, respectively. 4

**Figure S3**. Comparison of HPLC-DAD/MS profiles of strains cultivated in YM6.3 media. Relative absorbance at 210 nm is shown. A) crude extracts from mycelia, B) crude extracts from supernatant. Crude extracts form plant-associated and nematode‑associated strains are indicated in red and black, respectively. 5

**Figure S4.** Bayesian inference of phylogenetic relationships using six strains of the fungus described here based on an alignment of ITS, LSU, SSU, rpb2 and tef1 sequences using GTR+I+G as nucleotide substitution model. Depicted is a 50% majority rule consensus tree derived from 7500 trees from the stationary phase of a Monte Carlo Markov Chain. A posteriori probability (BIpp) values greater than 0.90, and bootstrap values of maximum likelihood (MLBT) and neighbor-joining (NJBT) analyses greater than 0.7 are given above branches (BIpp/MLBT/NJBT). The tree was rooted as midpoint. 6

[**Table S1** Table of taxa. Isolates and accession numbers used for the five-marker phylogeny 7](#_Toc123393385)

[**Table S2.** Comparison of metabolite occurrence in the HPLC-DAD/MS profiles of strains cultivated in different media including Q6/2,YM6.3, ZM/2, and seperated between mycelia (M) and supernatant (S) extracts.* indicates metabolites published by Helaly et al. 2018: (*1 ophiotine; *2 xanthomide Z; *3 arthrichitin; 4* arthrichitin B; 5* arthrichitin C). + indicates compounds detected in minor or large amounts, (+) indicates compounds detected in traces or beneath other signals (verified by mass spectrometry). Numbers refers to the Figs 2, S2 & S3. 9](#_Toc123393386)


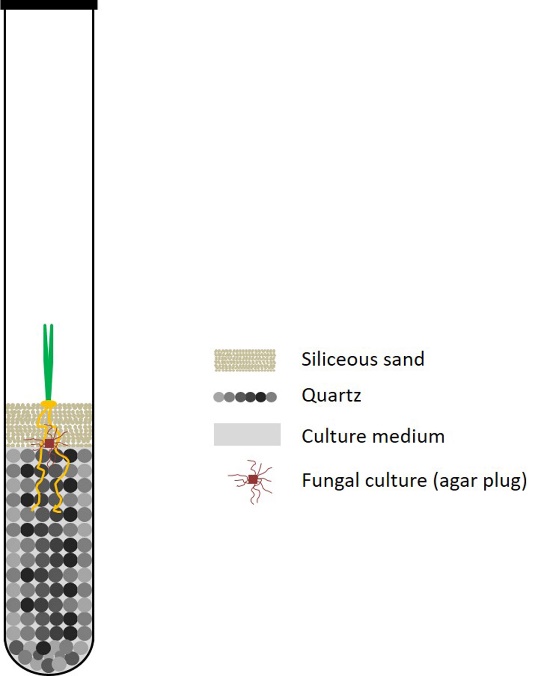


**Figure S1.** Schematic overview of the axenic glass tube system to demonstrate fungal colonization process and potential structural changes in root cells. Tube were filled by quartz and MS culture medium.


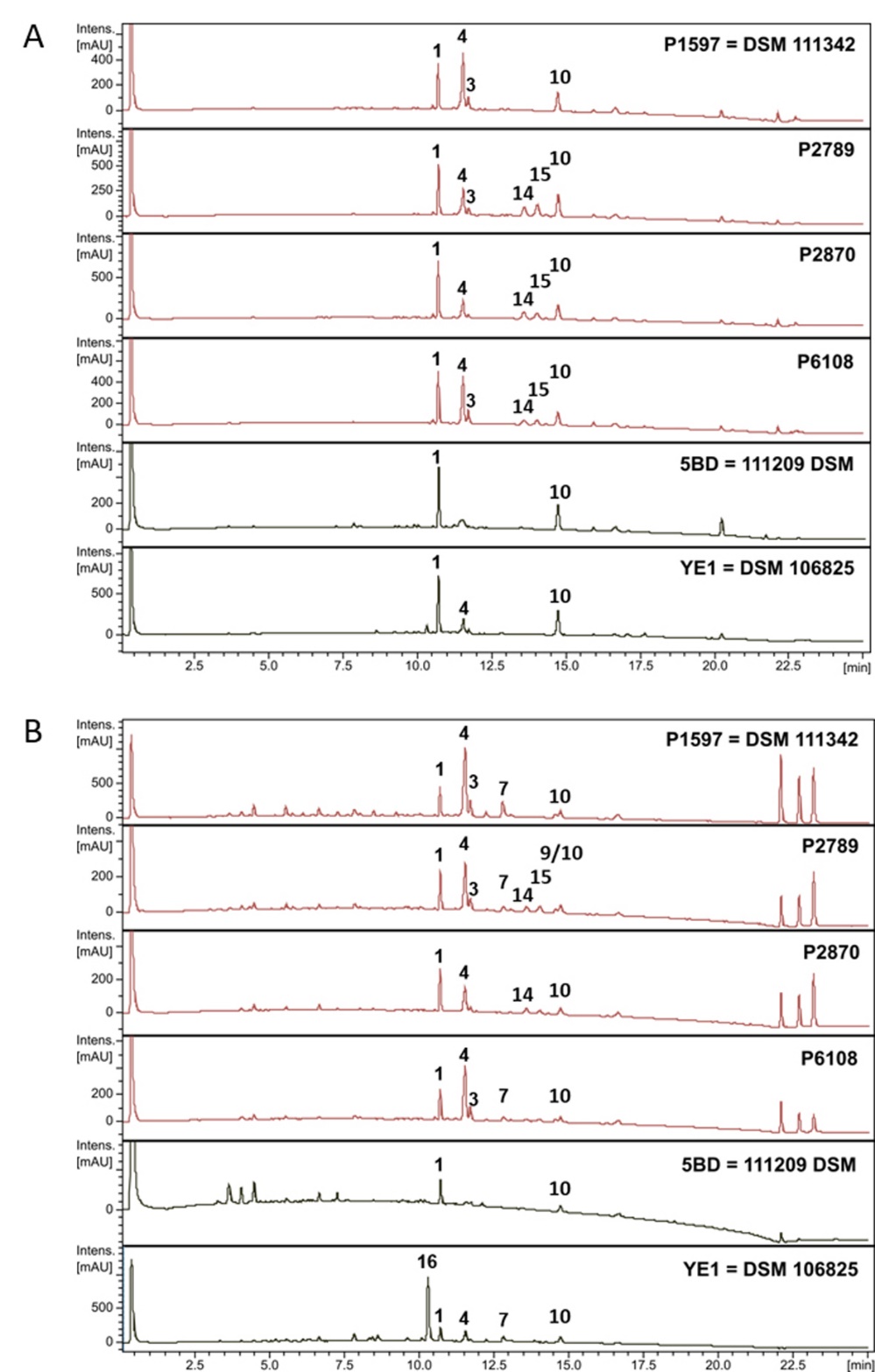


**Figure S2.** Comparison of HPLC-DAD/MS profiles of strains cultivated in Q6/2 media. Relative absorbance at 210 nm is shown. A) crude extracts from mycelia, B) crude extracts from supernatant. Crude extracts form plant-associated and nematode‑associated strains are indicated in red and black, respectively.


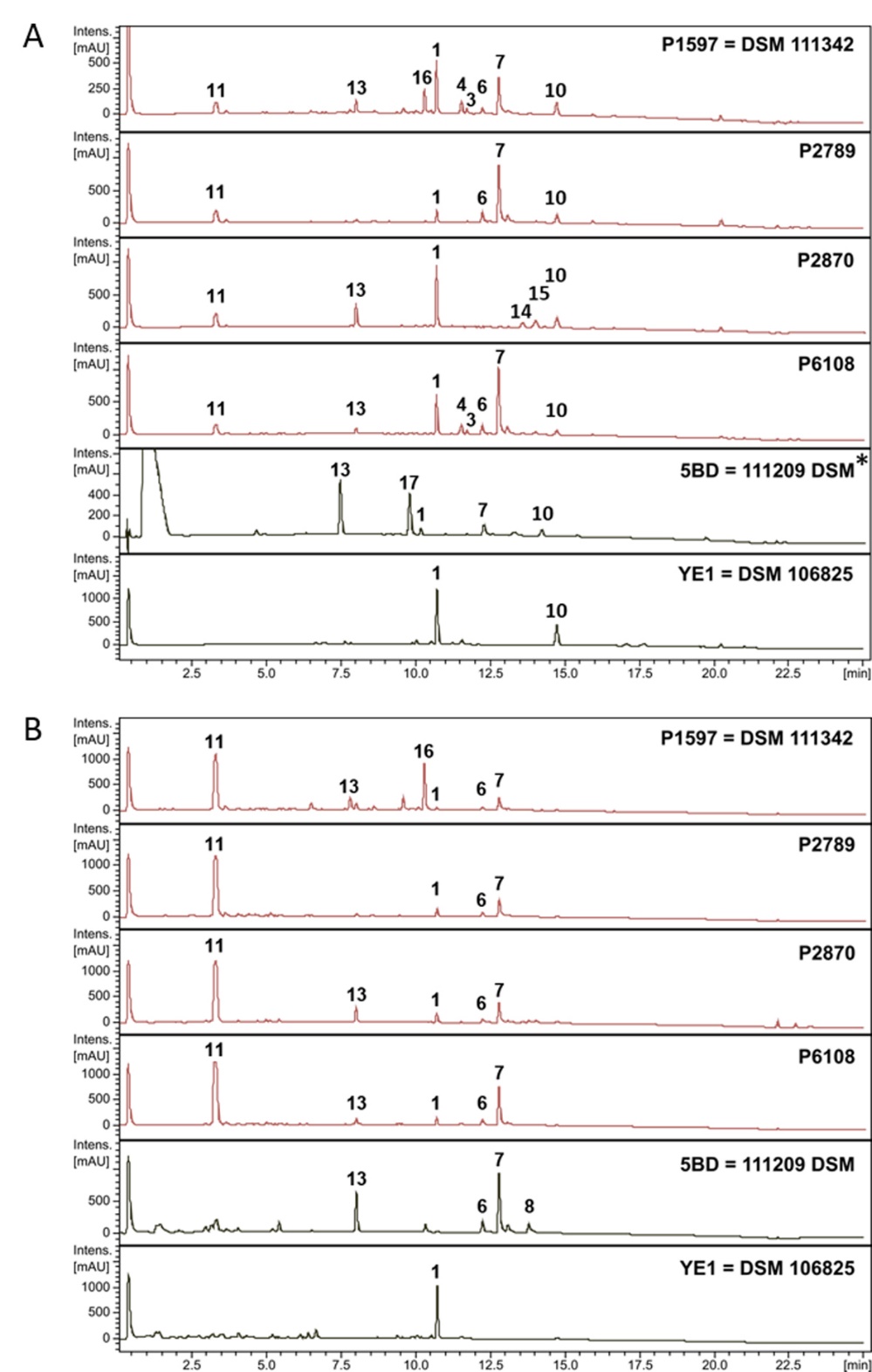


**Figure S3**. Comparison of HPLC-DAD/MS profiles of strains cultivated in YM6.3 media. Relative absorbance at 210 nm is shown. A) crude extracts from mycelia, B) crude extracts from supernatant. Crude extracts form plant-associated and nematode‑associated strains are indicated in red and black, respectively.


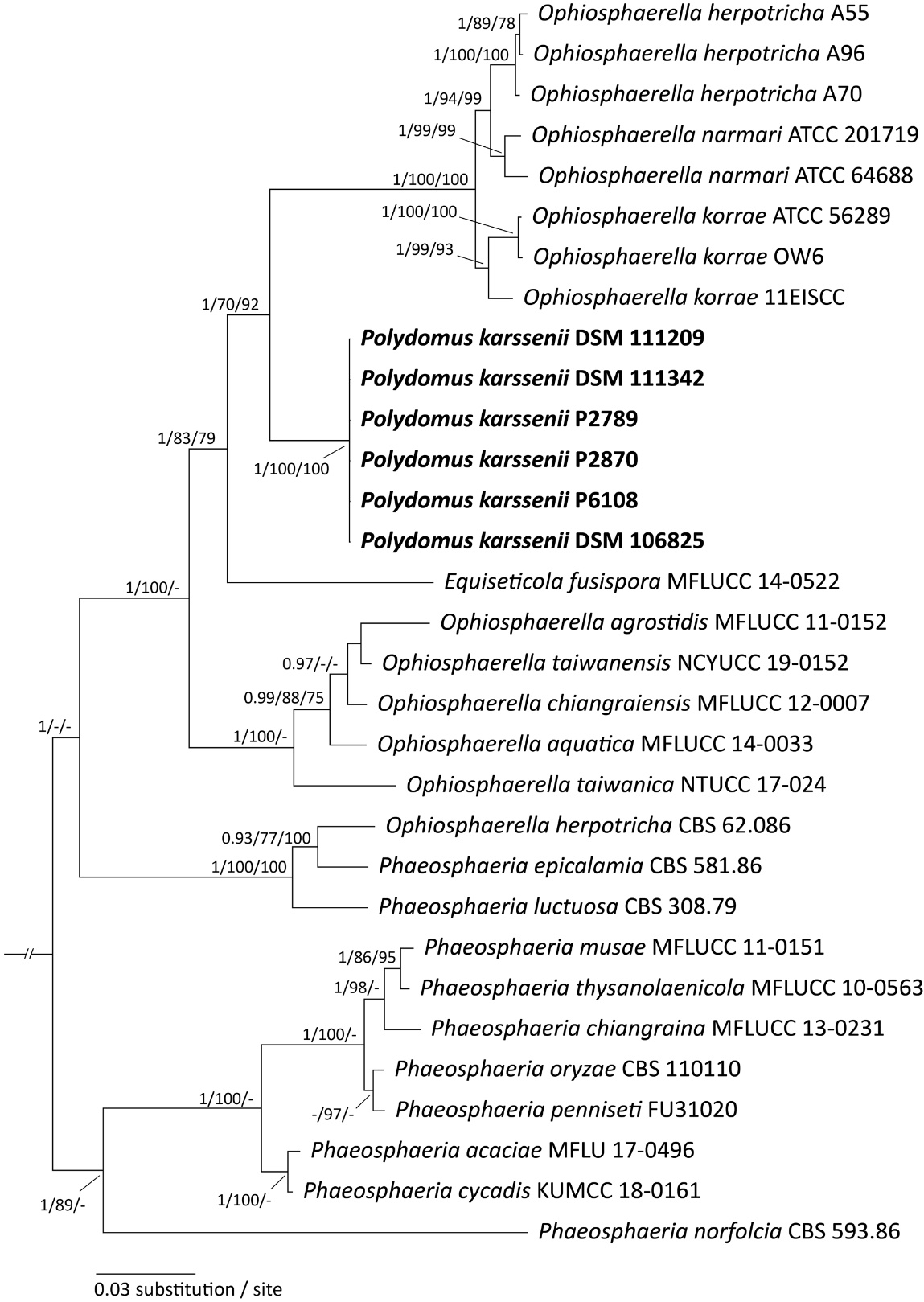


**Figure S4.** Bayesian inference of phylogenetic relationships using six strains of the fungus described here based on an alignment of ITS, LSU, SSU, rpb2 and tef1 sequences using GTR+I+G as nucleotide substitution model. Depicted is a 50% majority rule consensus tree derived from 7500 trees from the stationary phase of a Monte Carlo Markov Chain. A posteriori probability (BIpp) values greater than 0.90, and bootstrap values of maxi­mum likelihood (MLBT) and neighbor-joining (NJBT) analyses greater than 0.7 are given above branches (BIpp/MLBT/NJBT). The tree was rooted as midpoint.

**Table S1** Table of taxa. Isolates and accession numbers used for the five-marker phylogeny

| Species | strain number | Host / substrate | Locality | GenBank accession numbers | | | | | Reference |
| --- | --- | --- | --- | --- | --- | --- | --- | --- | --- |
|  |  |  |  | ITS | LSU | SSU | *rpb2* | *tef1* |  |
| *Equiseticola_fusispora* | MFLUCC 14-0522 | *Equisetum sp.* | Italy | NR_154010 | NG059249 | NG061238 | courtesy of Rungtiwa Phookamsak | MG520895 | (Abd-Elsalam et al. 2016) |
| *Ophiosphaerella_agrostidis* | MFLUCC 11-0152 | dead culms, grasses | Thailand | KM434271 | KM434281 | KM434290 | - | KM434299 | (Phookamsak et al. 2017) |
| *Ophiosphaerella_aquatica* | MFLUCC 14-0033 | decaying submerged wood | Thailand | NR_154352 | KX767089 | NG063623 | - | MG520911 | (Ariyawansa et al. 2015) |
| *Ophiosphaerella_chiangraiensis* | MFLUCC 12-0007 | dead culms, grasses | Thailand | KM434272 | KM434282 | KM434291 | KM434308 | KM434300 | (Yuan et al. 2020) |
| *Ophiosphaerella_herpotricha* | A55 | Bermudagrass | Stillwater, OK | KC841046 | KC841046 | KC841046 | KC840983 | KC841109 | (Flores et al. 2017) |
| *Ophiosphaerella_herpotricha* | A70 | Bermudagrass | Afton, OK | KC841052 | KC841052 | KC841052 | KC840989 | KC841115 | (Flores et al. 2017) |
| *Ophiosphaerella_herpotricha* | A96 | Bermudagrass | Oklahoma City, OK | KC841059 | KC841059 | KC841059 | KC840996 | KC841122 | (Flores et al. 2017) |
| *Ophiosphaerella_herpotricha* | CBS 620.86 /AFTOL-ID 1569 | *Bromus erectus* | Switzerland | ON407117 | ON407080 | ON408351 | ON419516 | ON419505 | This study |
| *Ophiosphaerella_korrae* | 11EISCC | Bermudagrass | Tulsa, OK | KC841021 | KC841021 | KC841021 | KC840958 | KC841084 | (Flores et al. 2017) |
| *Ophiosphaerella_korrae* | ATCC56289 | Kentucky bluegrass | New York | KC848509 | KC848509 | KC848509 | KC848512 | KC848515 | (Flores et al. 2017) |
| *Ophiosphaerella_korrae* | OW6 | Bermudagrass | West Point, MS | KC841077 | KC841077 | KC841077 | KC841014 | KC841140 | (Flores et al. 2017) |
| *Ophiosphaerella_narmari* | ATCC 201719 | Bermudagrass | Afton, OK | KC848508 | KC848508 | KC848508 | KC848511 | KC848514 | (Flores et al. 2017) |
| *Ophiosphaerella_narmari* | ATCC 64688 | Bermudagrass | Australia | KC848510 | KC848510 | KC848510 | KC848513 | KC848516 | (Flores et al. 2017) |
| *Ophiosphaerella_taiwanensis* | NCYUCC 19-0152 | *Agave tequilana* | Taiwan | MT321801 | MT321815 | MT321808 | - | MT328758 | (Tennakoon et al. 2020) |
| *Ophiosphaerella_taiwanica* | NTUCC 17-024 | dead stems of *Yushania niitakayamensis* | Taiwan | MN082417 | MN082419 | - | - | MN199124 | (Ariyawansa and Jones 2019) |
| *Phaeosphaeria_acaciae* | MFLU 17-0496 | *Acasia* sp. | China | NR_160335 | NG069453 | NG065678 | - | - | GenBank |
| *Phaeosphaeria_chiangraina* | MFLUCC 13-0231 | dead branches | Thailand | NR_155643 | NG069237 | NG063560 | KM434307 | KM434298 | (Phookamsak et al. 2017) |
| *Phaeosphaeria_cycadis* | KUMCC 18-0161 | *Cycas* sp. | China | NR_164445 | NG070078 | NG067700 | - | MK359069 | GenBank |
| *Phaeosphaeria_epicalamia* | CBS 581.86 | *Luzula sylvatica* | Switzerland | ON407118 | ON407081 | ON408352 | ON419517 | ON419506 | This study |
| *Phaeosphaeria_luctuosa* | CBS 308.79 | *Zea* *mays* | Switzerland | ON407119 | ON407082 | ON408353 | ON419518 | ON419507 | This study |

**Table S1** Continued

| Species | strain number | Host / substrate | Locality | GenBank accession numbers | | | | | Reference |
| --- | --- | --- | --- | --- | --- | --- | --- | --- | --- |
|  |  |  |  | ITS | LSU | SSU | *rpb2* | *tef1* |  |
| *Phaeosphaeria_musae* | MFLUCC 11-0151 | *Cordyline* sp. | Thailand | KM434268 | KM434278 | KM434288 | KM434305 | KM434297 | (Phookamsak et al. 2017) |
| *Phaeosphaeria_norfolcia* | CBS 593.86 | *Juncus inflexus* | Switzerland | ON407120 | ON407083 | ON408354 | ON419519 | ON419508 | This study |
| *Phaeosphaeria_oryzae* | CBS 110110 | *Oryza sativa* | Korea | ON407121 | ON407084 | ON408355 | ON419520 | ON419509 | This study |
| *Phaeosphaeria_penniseti* | FU31020 | dead culms | Taiwan | MK503819 | MK503825 | MK503831 | - | - | (Phookamsak et al. 2014) |
| *Phaeosphaeria_thysanolaenicola* | MFLUCC 10-0563 | *Thysanolaena maxima* | Thailand | NR_155642 | NG069236 | NG063559 | KM434303 | KM434295 | (Phookamsak et al. 2017) |
| *Polydomus karssenii* | YE1 = DSM106825 = JKI 72994 | Eggs of *Heterodera filipjevi* | Turkey | ON407111 | ON407074 | ON408345 | ON419510 | ON419499 | This study |
| *Polydomus karssenii* | 5BD = DSM111209 = JKI 73116 | Eggs of *Heterodera filipjevi* | Turkey | ON407112 | ON407075 | ON408346 | ON419511 | ON419500 | This study |
| *Polydomus karssenii* | P1597 = DSM111342 = JKI 73117 | *Microthlaspi perfoliatum* | Bulgaria | ON407113 | ON407076 | ON408347 | ON419512 | ON419501 | This study |
| *Polydomus karssenii* | P2789 = JKI 73120 | *Microthlaspi perfoliatum* | Germany | ON407114 | ON407077 | ON408348 | ON419513 | ON419502 | This study |
| *Polydomus karssenii* | P2870 = JKI 73119 | *Microthlaspi perfoliatum* | Germany | ON407115 | ON407078 | ON408349 | ON419514 | ON419503 | This study |
| *Polydomus karssenii* | P6108 = JKI 73118 | *Microthlaspi perfoliatum* | Germany | ON407116 | ON407079 | ON408350 | ON419515 | ON419504 | This study |

**ATCC**: American Type Culture Collection, Manassas, VA, USA; **CBS**: Westerdijk Fungal Biodiversity Institute, Utrecht, The Netherlands; **DSM**: The open collection of the Leibniz-Institut DSMZ- Deutsche Sammlung von Mikroorganismen und Zellkulturen GmbH; Germany; **MFLU, MFLUCC**: Mae Fah Luang University Culture Collection; Thailand; JKI: The fungal collection of the Julius Kuehn Institute, Germany.

**Table S2.** Comparison of metabolite occurrence in the HPLC-DAD/MS profiles of strains cultivated in different media including Q6/2,YM6.3, ZM/2, and seperated between mycelia (M) and supernatant (S) extracts.* indicates metabolites published by Helaly et al. 2018: (*1 ophiotine; *2 xanthomide Z; *3 arthrichitin; 4* arthrichitin B; 5* arthrichitin C). + indicates compounds detected in minor or large amounts, (+) indicates compounds detected in traces or beneath other signals (verified by mass spectrometry). Numbers refers to the Figs 2, S2 & S3.

| Species | Media | extract | 1* | 2* | 3* | 4* | 5* | 6 | 7 | 8 | 9 | 10 | 11 | 12 | 13 | 14 | 15 | 16 | 17 |
| --- | --- | --- | --- | --- | --- | --- | --- | --- | --- | --- | --- | --- | --- | --- | --- | --- | --- | --- | --- |
| P1579  73117 | Q6/2 | M | + |  | + | + | (+) | (+) | (+) | (+) | (+) | + |  |  |  |  |  |  |  |
|  |  | S | + |  | + | + | (+) | (+) | + | (+) | (+) | + | (+) |  |  |  |  | (+) |  |
|  | YM 6.3 | M | + | (+) | (+) | + |  | (+) | + | (+) | (+) | + | + |  | + |  |  | + |  |
|  |  | S | (+) |  | (+) | (+) |  | + | + | (+) |  | (+) | + |  | + |  |  | + |  |
|  | ZM/2 | M | + |  | + | + |  | + | + | + | (+) | (+) | (+) |  | (+) |  |  | (+) |  |
|  |  | S | (+) |  | (+) | + |  | + | + | + | + | (+) | + |  | (+) | (+) | (+) |  |  |
| P2789  73120 | Q6/2 | M | + |  | + | + | (+) | (+) | (+) | (+) | (+) | + |  |  | (+) | + | + |  |  |
|  |  | S | + |  | + | + |  | (+) | + | (+) | + | + | (+) |  | (+) | + | + |  |  |
|  | YM 6.3 | M | + |  | (+) | (+) |  | + | + | (+) | (+) | + | + |  | (+) | (+) | (+) | (+) | (+) |
|  |  | S | + |  | (+) | (+) |  | + | + |  |  | (+) | + |  | (+) | (+) | (+) |  |  |
|  | ZM/2 | M | + |  | + | + |  | + | + | + | (+) | + | (+) |  | + | (+) | + | (+) | (+) |
|  |  | S | (+) | (+) | (+) | + |  | + | + | + | + | (+) | (+) |  | + | (+) | (+) |  |  |
| P2870  73119 | Q6/2 | M | + | (+) | (+) | (+) |  |  | (+) |  |  | + | (+) |  | (+) | + | + |  |  |
|  |  | S | + |  | (+) | + |  |  | (+) |  |  | + | (+) |  | (+) | + | + |  |  |
|  | YM 6.3 | M | + | (+) | (+) | (+) |  | (+) | (+) | (+) |  | + | + |  | + | + | + |  | (+) |
|  |  | S | + |  | (+) | (+) |  | + | + | (+) |  | (+) | + |  | + | (+) | (+) |  |  |
|  | ZM/2 | M | + |  | (+) | + |  | + | + | + | (+) | (+) | (+) |  | (+) | (+) | (+) |  | (+) |
|  |  | S | (+) | (+) | (+) | (+) |  | + | + | + | + | (+) | (+) |  | + | (+) | (+) |  |  |
| P6108  73118 | Q6/2 | M | + |  | + | + |  | (+) | (+) | (+) | (+) | + |  |  | (+) | + | + |  |  |
|  |  | S | + |  | + | + |  | (+) | + | (+) | (+) | + | (+) |  | (+) | (+) | (+) |  |  |
|  | YM 6.3 | M | + |  | + | + | (+) | + | + | (+) |  | + | + |  | + | (+) | (+) |  | (+) |
|  |  | S | + |  | (+) | (+) |  | + | + | (+) |  | (+) | + |  | + | (+) | (+) |  | (+) |
|  | ZM/2 | M | + | (+) | + | + |  | + | + | + | (+) | + | (+) |  | + | (+) | (+) |  | (+) |
|  |  | S | (+) | + | (+) | + |  | + | + | (+) | (+) | + | (+) |  | + | (+) | (+) |  |  |
| 5BD | Q6/2 | M | + |  | (+) | (+) |  |  |  |  |  | + | (+) |  |  |  |  |  |  |
|  |  | S | + |  | (+) | (+) |  |  |  |  |  | + | (+) |  |  |  |  |  |  |
|  | YM 6.3 | M | + |  | (+) | (+) |  | (+) | + | (+) |  | + |  |  | + |  |  |  | + |
|  |  | S | (+) | (+) | (+) | (+) |  | + | + | + |  | (+) | (+) |  | + |  |  |  | + |
|  | ZM/2 | M | + | + | (+) | + |  | + | + | + | (+) | + |  |  | (+) |  |  |  | (+) |
|  |  | S | + | + | (+) | (+) |  | + | + | + | (+) | + | (+) |  | + |  |  |  |  |
| YE1 | Q6/2 | M | + | (+) | + | + |  | (+) | (+) | (+) | (+) | + |  |  |  |  |  | (+) |  |
|  |  | S | + | (+) | (+) | + |  | (+) | + | (+) | (+) | + | (+) |  |  |  |  | + |  |
|  | YM 6.3 | M | + | (+) | (+) | + |  |  | (+) |  |  | + | (+) |  |  |  |  | (+) |  |
|  |  | S | + | (+) | (+) | (+) |  |  | (+) |  |  | (+) | (+) |  |  |  |  |  |  |
|  | ZM/2 | M | + | + | (+) | + |  | (+) | + | (+) | (+) | + | (+) | (+) |  |  |  |  |  |
|  |  | S | + | + | (+) | + |  | (+) | + | (+) | (+) | (+) | (+) | + |  |  |  |  |  |

# References

Abd-Elsalam KA, Tibpromma S, Wanasinghe DN, Camporesi E, Hyde KD (2016) *Equiseticola* gen. nov. (Phaeosphaeriaceae), from *Equisetum* sp. in Italy. Phytotaxa 284.

Ariyawansa HA, Hyde KD, Jayasiri SC, Buyck B, Chethana KWT, Dai DQ, Dai YC, Daranagama DA, Jayawardena RS, Lücking R, Ghobad-Nejhad M, Niskanen T, Thambugala KM, Voigt K, Zhao RL, Li G-J, Doilom M, Boonmee S, Yang ZL, Cai Q, Cui Y-Y, Bahkali AH, Chen J, Cui BK, Chen JJ, Dayarathne MC, Dissanayake AJ, Ekanayaka AH, Hashimoto A, Hongsanan S, Jones EBG, Larsson E, Li WJ, Li Q-R, Liu JK, Luo ZL, Maharachchikumbura SSN, Mapook A, McKenzie EHC, Norphanphoun C, Konta S, Pang KL, Perera RH, Phookamsak R, Phukhamsakda C, Pinruan U, Randrianjohany E, Singtripop C, Tanaka K, Tian CM, Tibpromma S, Abdel-Wahab MA, Wanasinghe DN, Wijayawardene NN, Zhang J-F, Zhang H, Abdel-Aziz FA, Wedin M, Westberg M, Ammirati JF, Bulgakov TS, Lima DX, Callaghan TM, Callac P, Chang C-H, Coca LF, Dal-Forno M, Dollhofer V, Fliegerová K, Greiner K, Griffith GW, Ho H-M, Hofstetter V, Jeewon R, Kang JC, Wen T-C, Kirk PM, Kytövuori I, Lawrey JD, Xing J, Li H, Liu ZY, Liu XZ, Liimatainen K, Lumbsch HT, Matsumura M, Moncada B, Nuankaew S, Parnmen S, de Azevedo Santiago ALCM, Sommai S, Song Y, de Souza CAF, de Souza-Motta CM, Su HY, Suetrong S, Wang Y, Wei S-F, Wen TC, Yuan HS, Zhou LW, Réblová M, Fournier J, Camporesi E, Luangsa-ard JJ, Tasanathai K, Khonsanit A, Thanakitpipattana D, Somrithipol S, Diederich P, Millanes AM, Common RS, Stadler M, Yan JY, Li X, Lee HW, Nguyen TTT, Lee HB, Battistin E, Marsico O, Vizzini A, Vila J, Ercole E, Eberhardt U, Simonini G, Wen H-A, Chen X-H, Miettinen O, Spirin V, Hernawati (2015) Fungal diversity notes 111–252—taxonomic and phylogenetic contributions to fungal taxa. Fungal Diversity 75: 27-274.

Ariyawansa HA, Jones EBG (2019) Additions to Taiwan fungal flora 2: *Ophiosphaerella taiwanica* sp. nov. Phytotaxa 413: 39-48.

Flores FJ, Marek SM, Orquera G, Walker NR (2017) Molecular identification and multilocus phylogeny of *Ophiosphaerella* species associated with spring dead spot of Bermudagrass. Crop Science 57.

Phookamsak R, Liu J-K, McKenzie EHC, Manamgoda DS, Ariyawansa H, Thambugala KM, Dai D-Q, Camporesi E, Chukeatirote E, Wijayawardene NN, Bahkali AH, Mortimer PE, Xu J-C, Hyde KD (2014) Revision of Phaeosphaeriaceae. Fungal Diversity 68: 159-238.

Phookamsak R, Wanasinghe DN, Hongsanan S, Phukhamsakda C, Huang S-K, Tennakoon DS, Norphanphoun C, Camporesi E, Bulgakov TS, Promputtha I, Mortimer PE, Xu J-C, Hyde KD (2017) Towards a natural classification of *Ophiobolus* and *ophiobolus*-like taxa; introducing three novel genera *Ophiobolopsis*, *Paraophiobolus* and *Pseudoophiobolus* in Phaeosphaeriaceae (Pleosporales). Fungal Diversity 87: 299-339.

Tennakoon DS, Thambugala KM, Wanasinghe DN, Gentekaki E, Promputtha I, Kuo C-H, Hyde KD (2020) Additions to Phaeosphaeriaceae (Pleosporales): *Elongaticollum* gen. nov., *Ophiosphaerella taiwanensis* sp. nov., *Phaeosphaeriopsis beaucarneae* sp. nov. and a new host record of *Neosetophoma poaceicola* from Musaceae. MycoKeys 70: 59-88.

Yuan H-S, Lu X, Dai Y-C, Hyde KD, Kan Y-H, Kušan I, He S-H, Liu N-G, Sarma VV, Zhao C-L, Cui B-K, Yousaf N, Sun G, Liu S-Y, Wu F, Lin C-G, Dayarathne MC, Gibertoni TB, Conceição LB, Garibay-Orijel R, Villegas-Ríos M, Salas-Lizana R, Wei T-Z, Qiu J-Z, Yu Z-F, Phookamsak R, Zeng M, Paloi S, Bao D-F, Abeywickrama PD, Wei D-P, Yang J, Manawasinghe IS, Harishchandra D, Brahmanage RS, de Silva NI, Tennakoon DS, Karunarathna A, Gafforov Y, Pem D, Zhang S-N, de Azevedo Santiago ALCM, Bezerra JDP, Dima B, Acharya K, Alvarez-Manjarrez J, Bahkali AH, Bhatt VK, Brandrud TE, Bulgakov TS, Camporesi E, Cao T, Chen Y-X, Chen Y-Y, Devadatha B, Elgorban AM, Fan L-F, Du X, Gao L, Gonçalves CM, Gusmão LFP, Huanraluek N, Jadan M, Jayawardena RS, Khalid AN, Langer E, Lima DX, de Lima-Júnior NC, de Lira CRS, Liu J-K, Liu S, Lumyong S, Luo Z-L, Matočec N, Niranjan M, Oliveira-Filho JRC, Papp V, Pérez-Pazos E, Phillips AJL, Qiu P-L, Ren Y, Ruiz RFC, Semwal KC, Soop K, de Souza CAF, Souza-Motta CM, Sun L-H, Xie M-L, Yao Y-J, Zhao Q, Zhou L-W (2020) Fungal diversity notes 1277–1386: taxonomic and phylogenetic contributions to fungal taxa. Fungal Diversity 104: 1-266.
